# Supplementary material for: Synergistic enzymatic and bioorthogonal reactions for selective prodrug activation in living systems
Source: Nat Commun. 2018 Nov 28;9:5032. doi: 10.1038/s41467-018-07490-6 (PMC6261997; doi:10.1038/s41467-018-07490-6)
Supplement: Supplementary file 1 — Supplementary information [file 41467_2018_7490_MOESM1_ESM.pdf]

# Supplementary Information

## Synergistic Enzymatic and Bioorthogonal Reactions for Selective Prodrug Activation in Living Systems

Qingxin Yao,[1,2] Feng Lin,[3,4] Xinyuan Fan,[4] Yanpu Wang,[5] Ye Liu,[1] Zhaofei Liu,[5] Xingyu Jiang,[1,2] Peng R. Chen[3,4,*] and Yuan Gao[1,2,*]

[1] CAS Center for Excellence in Nanoscience, CAS Key Laboratory of Biomedical Effects of Nanomaterials and Nanosafety, National Center for Nanoscience and Technology, Beijing 100190, China;

[2] University of Chinese Academy of Sciences, Beijing 100049, China;

[3] Key Laboratory of Bioorganic Chemistry and Molecular Engineering of Ministry of Education, College of Chemistry and Molecular Engineering, Peking University, Beijing 100871, China;

[4] Peking-Tsinghua Center for Life Sciences, Peking University, Beijing 100871, China;

[5] Medical Isotopes Research Center and Department of Radiation Medicine, School of Basic Medical Sciences, Peking University, Beijing 100191, China.

[*]E-mail: gaoy@nanoctr.cn; pengchen@pku.edu.cn

## Supplementary Figures

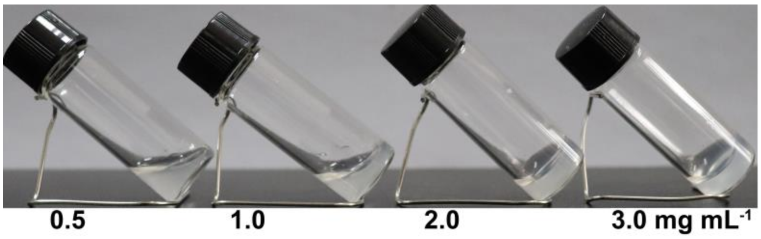


**Supplementary Figure 1. Optical images of 1 treated with alkaline phosphatase.** The critical hydrogelation concentration is 1.0 mg $mL^{-1}$. 10 mg $mL^{-1}$ stock solution of **1** was prepared by dissolving compound **1** in PBS and adjusting the solution pH to 7.4 using 1 mol $L^{-1}$ NaOH. Then, the stock solution was diluted to 0.5-3.0 mg $mL^{-1}$. To 0.5 mL solution at each concentration, 10 U $mL^{-1}$ of alkaline phosphatase was added. After 24 h, the hydrogel formed in 1.0-3.0 mg $mL^{-1}$.

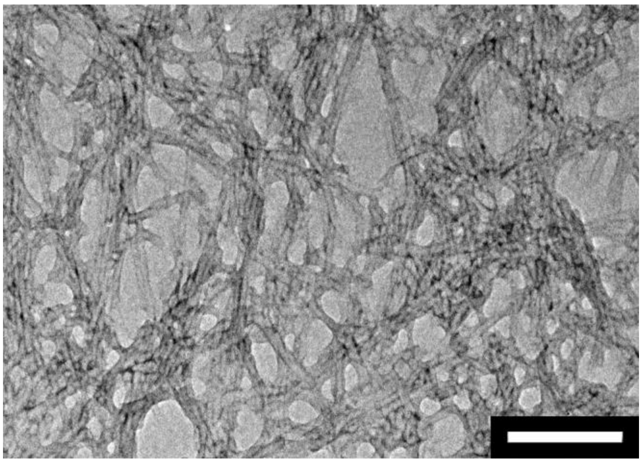

**Supplementary Figure 2. TEM image of nanofibres in hydrogel of 1.** TEM image showed there is a network of uniform nanofibres in the hydrogel of **1**. The hydrogel was prepared from 1.0 mg $mL^{-1}$ of **1** in PBS at pH 7.4 with the addition of 10 U $mL^{-1}$ of alkaline phosphatase. Scale bar, 200 nm.

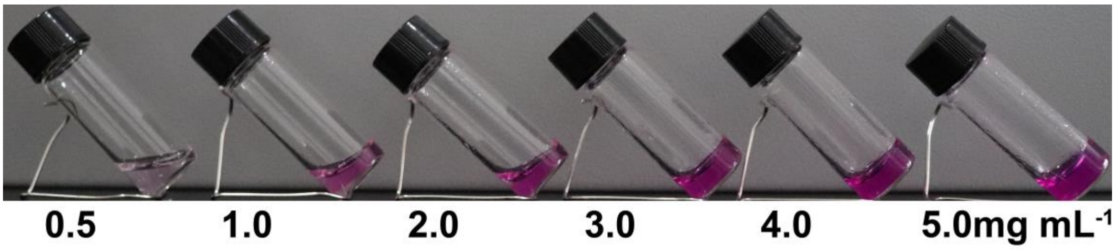


**Supplementary Figure 3. Optical images of 2 treated with alkaline phosphatase.** The critical hydrogelation concentration is 1.0 mg $mL^{-1}$. 10 mg $mL^{-1}$ stock solution of **2** was prepared by dissolving compound **2** in PBS and adjusting the solution pH to 7.4 using 1 mol $L^{-1}$ NaOH. Then, the stock solution was diluted to 0.5-5.0 mg $mL^{-1}$. To 0.5 mL solution at each concentration, 10 U $mL^{-1}$ of alkaline phosphatase was added. After 24 h, the hydrogel formed in 1.0-5.0 mg $mL^{-1}$.

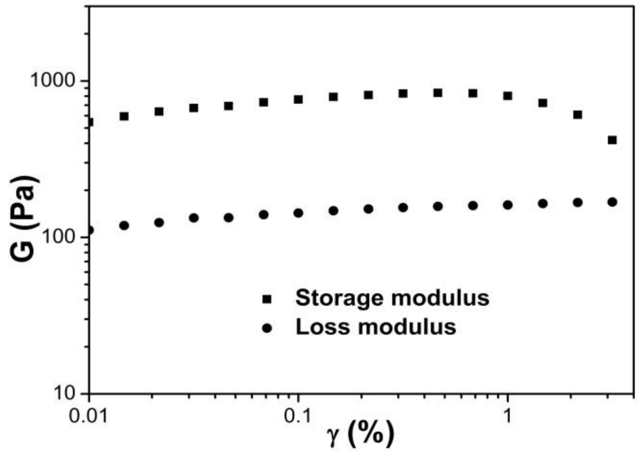


**Supplementary Figure 4**. **Strain sweep of the dynamic storage moduli (G') and the loss moduli (G'') of the hydrogel 3.** Hydrogel was prepared from 1.0 mg $mL^{-1}$ of **2** in PBS at pH 7.4 with the addition of 10 U $mL^{-1}$ of alkaline phosphatase for 24 h.

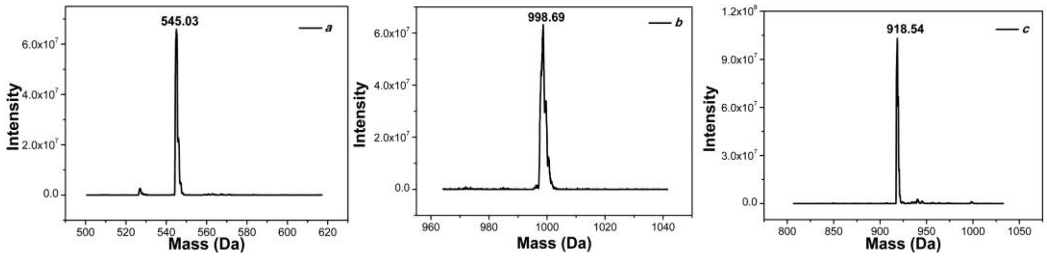


**Supplementary Figure 5. Mass spectra of peaks *a*, *b* and *c* shown in UPLC trace.** Peak *a* is doxorubicin, peak *b* is the inv-DA adduct, peak *c* is **2**.

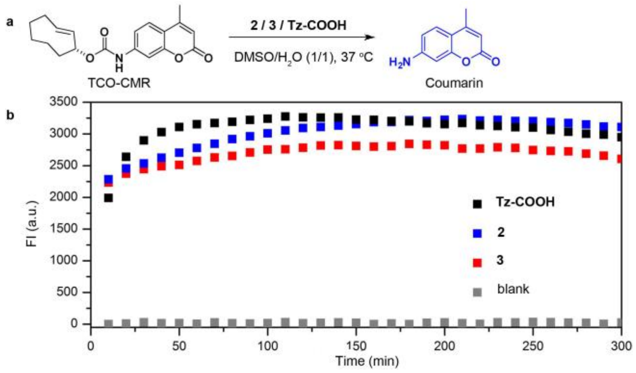


**Supplementary Figure 6**. **Swift coumarin release by 2, 3 or Tz-COOH.** A slightly lower decaging rate was observed for **3**, which may be attributed to the molecular arrangement that masked the activity of a small portion of tetrazine inside nanofibres.

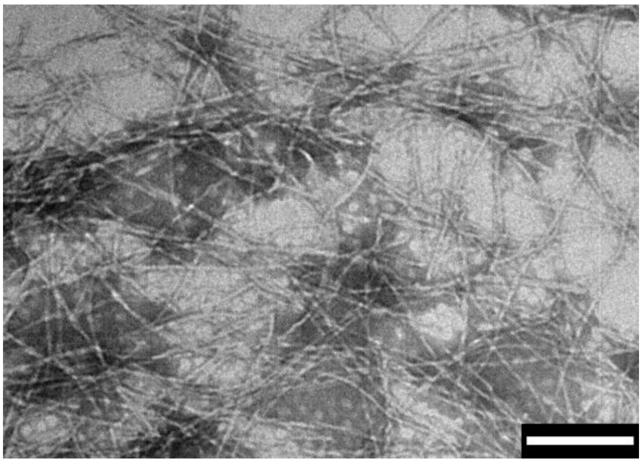

**Supplementary Figure 7. TEM image of hydrogel 3 with TCO adduct.** The morphology of nanofibres remained unchanged with TCO adduct after liberation of Dox. 0.5 mL of 1.0 mg mL$^{-1}$ hydrogel of **3** was mixed with 0.5 mL of 50 μM TCO-CMR in PBS for 24 h. Then the supernatant was removed and the hydrogel was inspected by TEM. Scale bar, 200 nm.

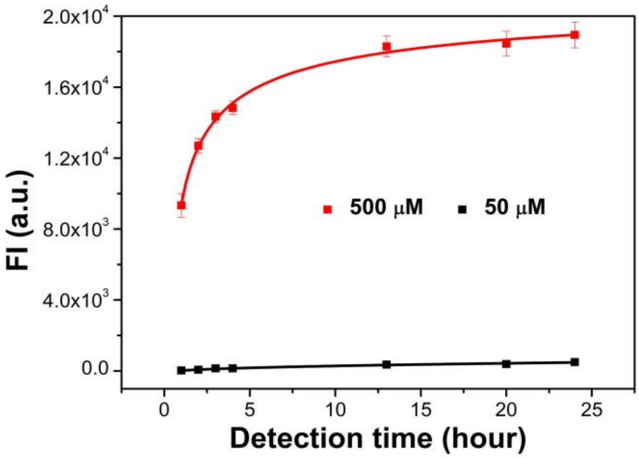


**Supplementary Figure 8. Time dependent development of fluorescence intensity by 2.** The total liberated fluorescence was proportional to the amount of **3** accumulated inside cells. Higher concentration of **2** favored the accumulation of **3** in HeLa cells. HeLa cells were pre-incubated with 100 μL of 50 or 500 μM **2** for 6h, then washed with PBS and incubated with 100 μL of 50 μM TCO-CMR for 1-24 h (n= 6). Error bars indicate standard deviation.

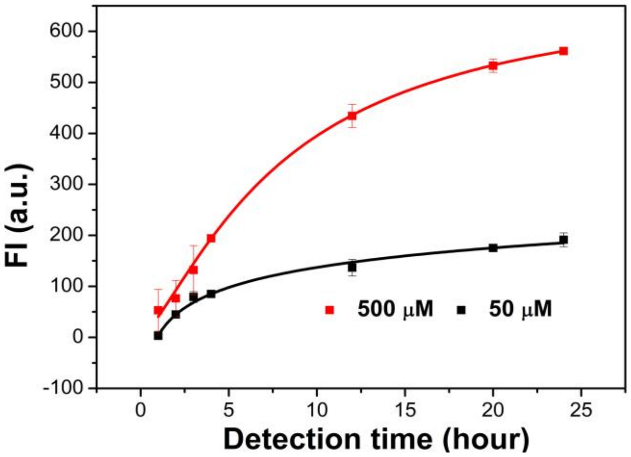


**Supplementary Figure 9. Time dependent development of weak fluorescence intensity by Tz-COOH.** Tz-COOH itself were hardly accumulated in HeLa cells, leading to weak liberated fluorescence. HeLa cells were pre-incubated with 100 μL of 50 or 500 μM Tz-COOH for 6 h, then washed with PBS and incubated with 100 μL of 50 μM TCO-CMR for 1-24 h (n= 6). Error bars indicate standard deviation.

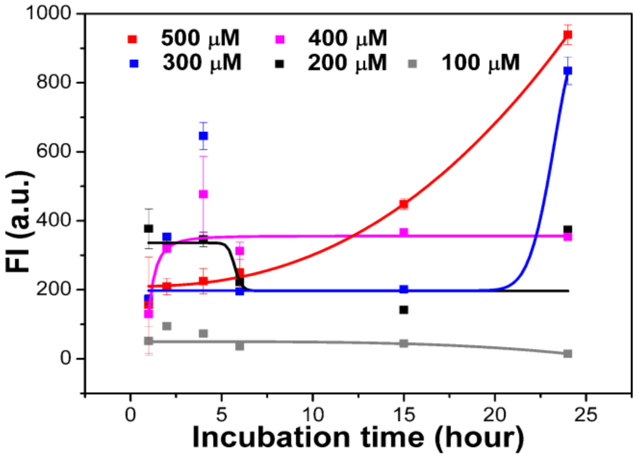


**Supplementary Figure 10. Weak fluorescence restored in HUVECs.** There is few/no self-assembly accumulated in HUVECs, leading to weak liberated fluorescence. HUVECs were pre-incubated with 100 μL of 100-500 μM **2** for 1-24 h, then washed with PBS and incubated with 100 μL of 50 μM TCO-CMR for 24 h (n= 6). Error bars indicate standard deviation.

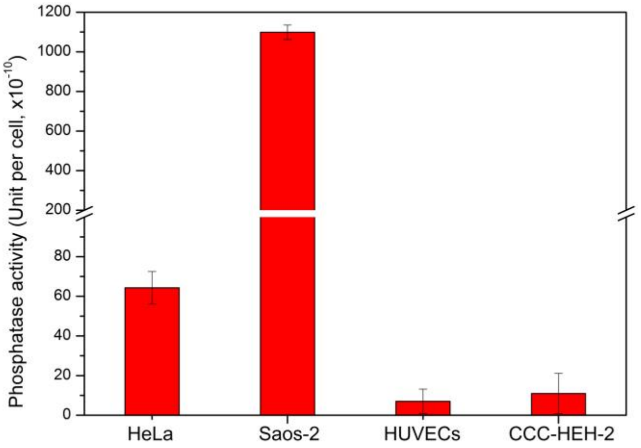


**Supplementary Figure 11. The phosphatase activity of different cell lines used in this study.** Cancer cell lines used in the study possess high phosphatase activity compared with normal cell lines. For each of the four cell lines (HeLa, Saos-2, HUVECs and CCC-HEH-2 cells), $3\times10^5$ cells were plated on a 6-well plate for 6 h before adding 150 μL of cell lysis buffer (1.0 mol $L^{-1}$ PMSF in RIPA, Beijing Solarbio Science and Technology Co., Ltd.). Cell samples were then centrifuged at 10000 g for 5 min and the supernatant was collected for the detection of phosphatase activity via a standard protocol using para-nitrophenyl phosphate (pNPP) as chromogenic substrate and para-nitrophenol as the reference (n= 6). Error bars indicate standard deviation.

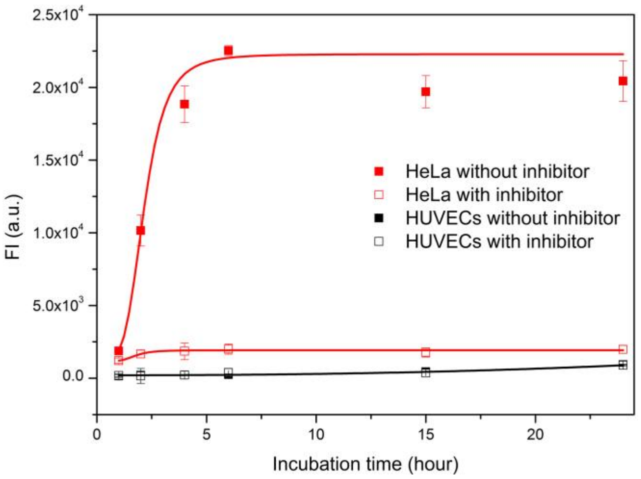


**Supplementary Figure 12. Effect of phosphatase inhibitor on the activation of TCO-CMR.** The addition of phosphatase inhibitor significantly decreased the accumulation of assembling molecules in HeLa cells, then influenced the activation of TCO-CMR. Cells were plated on flat bottom 96-well plates at a density of $10^4$ cells per well and allowed to attach for 6 h before being incubated with 100 μL of fresh culture medium w/o 25 μM phosphatase inhibitor cocktail (Beyotime Biothchnology) for 12 h. Cells were next treated with 100 μL of 500 μM **2** (w/o 25 μM phosphatase inhibitor cocktail) for 1, 2, 4, 6, 15 and 24 h, respectively. The culture medium was then removed, and cells were washed with PBS, incubated with 100 μL of 50 μM TCO-CMR for another 24 h. Finally, the fluorescence intensity of released coumarin was measured on a plate reader (n= 6). Error

bars indicate standard deviation.

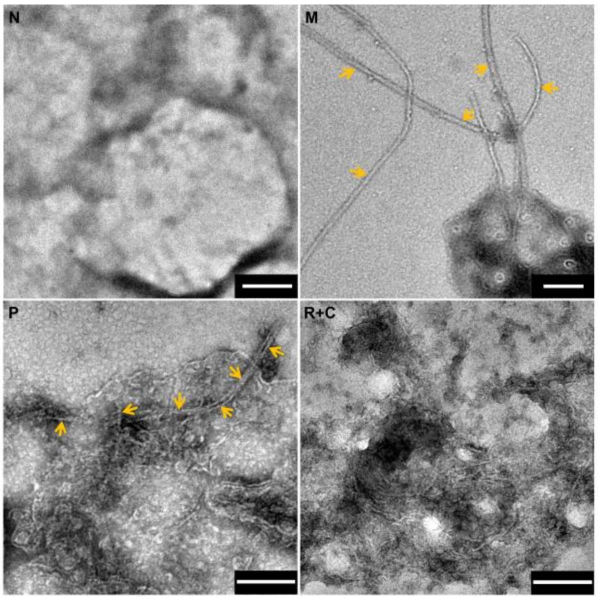


**Supplementary Figure 13. TEM images of cellular fractions of HeLa cells pre-incubated with 2.** Cells were pre-incubated with 500 μM **2** for 6 h and then subjected to cell lysis and fractionation.[7] The TEM images of the fractions **M** and **P** showed the existence of nanofibres, suggesting the formation of the nanofibres inside HeLa cells. Detailed protocol: HeLa cells were fractioned and divided into four parts, **N**, nuclei (scale bar, 500 nm); pellet sample **M**, mitochondria, lysosomes, peroxisomes (scale bar, 200 nm); pellet sample **P**, plasma membrane, microsomal fraction (fragments of ER), large polyribsomes (scale bar, 200 nm); pellet sample **R** and supernatant sample **C** (R+C), ribosomal subunits, small polyribosomes, soluble portion of cytoplasm (Cytosol, scale bar, 200 nm).

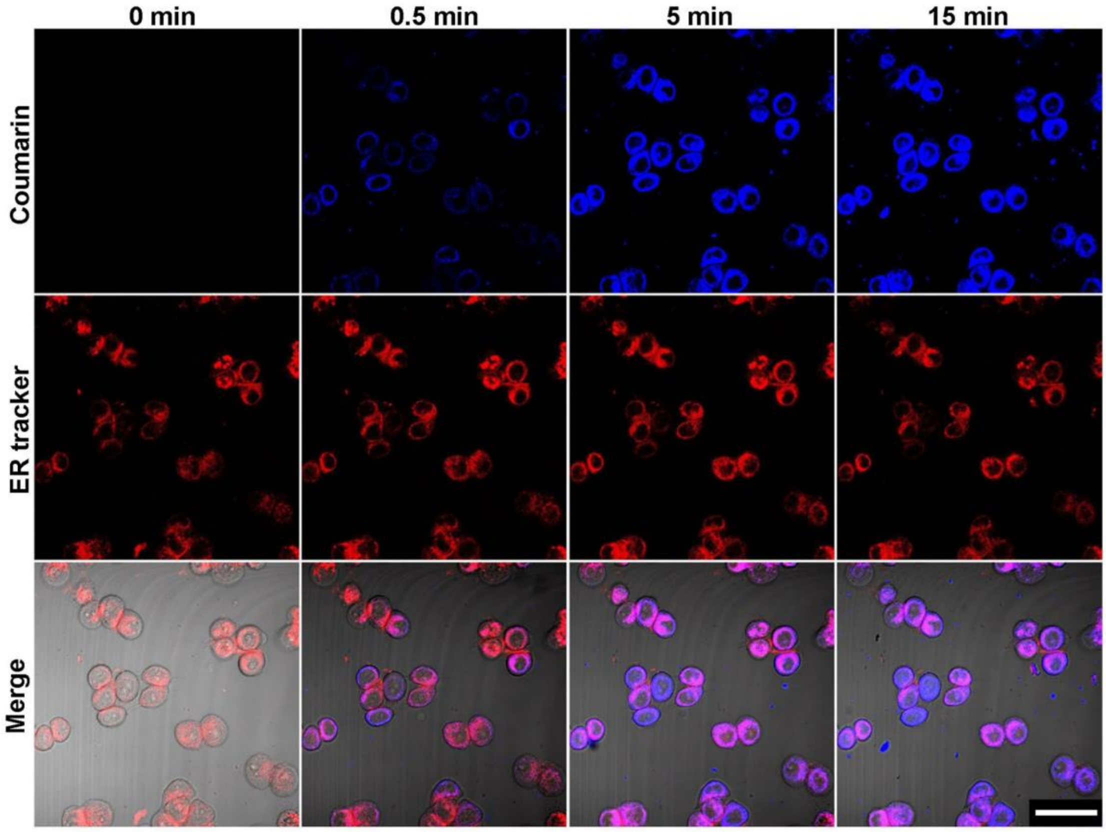


**Supplementary Figure 14. Time dependent confocal images in HeLa cells.** The images showed the development and distribution of liberated coumarin (blue) and its overlap with ER tracker (red) in HeLa cells. HeLa cells were pre-incubated with 2 mL culture medium containing 500 μM **2** for 6 h. Then, the cells were rinsed with PBS and cultured with 1 mL of 50 μM TCO-CMR for real time fluorescence imaging. ER was pre-stained by 500 nM ER-Tracker™ Red dyes before the addition of TCO-CMR. Scale bar, 50 μm.

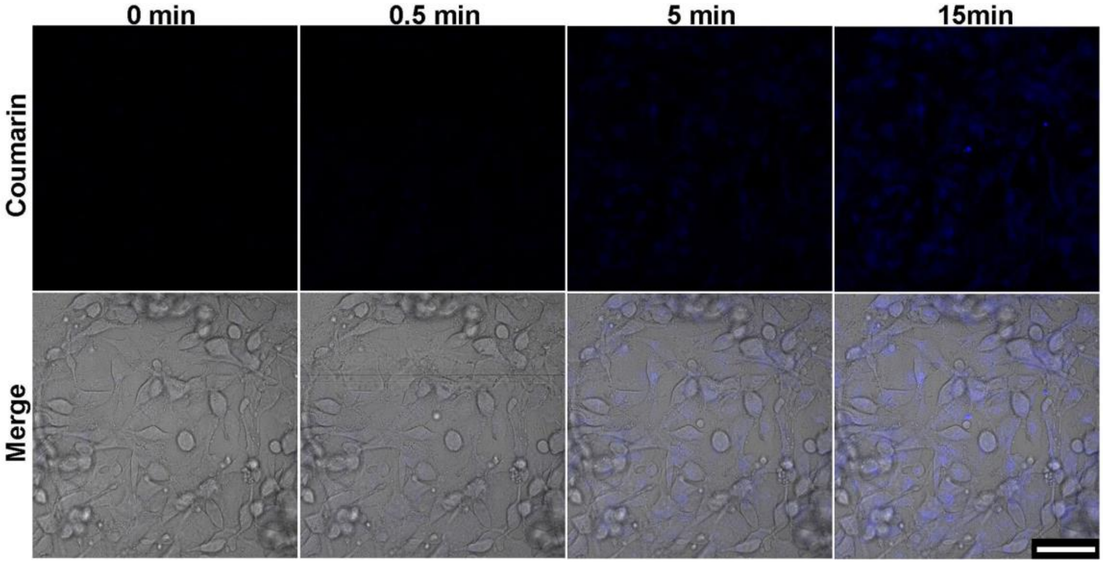


**Supplementary Figure 15. Time dependent confocal images in HUVECs.** The images showed the weak development of liberated coumarin (blue) in HUVECs. HUVECs were

pre-incubated with 2 mL culture medium containing 500 μM **2** for 6 h. Then, the cells were rinsed with PBS and incubated with 1 mL of 50 μM TCO-CMR for real time fluorescence imaging. Scale bar, 50 μm.

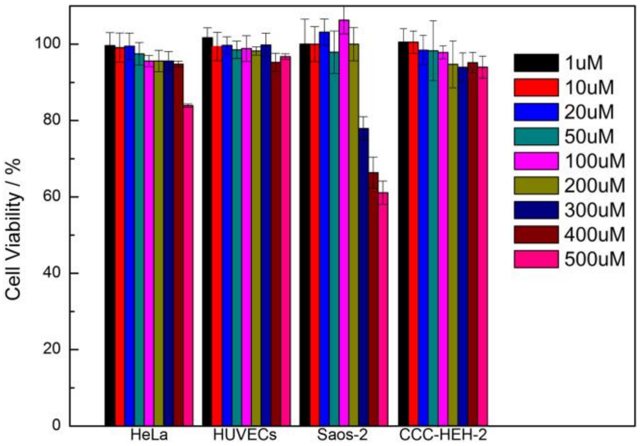


**Supplementary Figure 16. The 24 h cytotoxicity of 2 against different cell lines.** HeLa cells, HUVECs, Saos-2 cells and CCC-HEH-s cells at a density of $3x10^3$ cells per well were incubated with 100 μL pre-warmed medium containing 1-500 μM **2** for 24 h. Cell proliferation was then assessed by MTT assay (n= 6). Error bars indicate standard deviation.

.

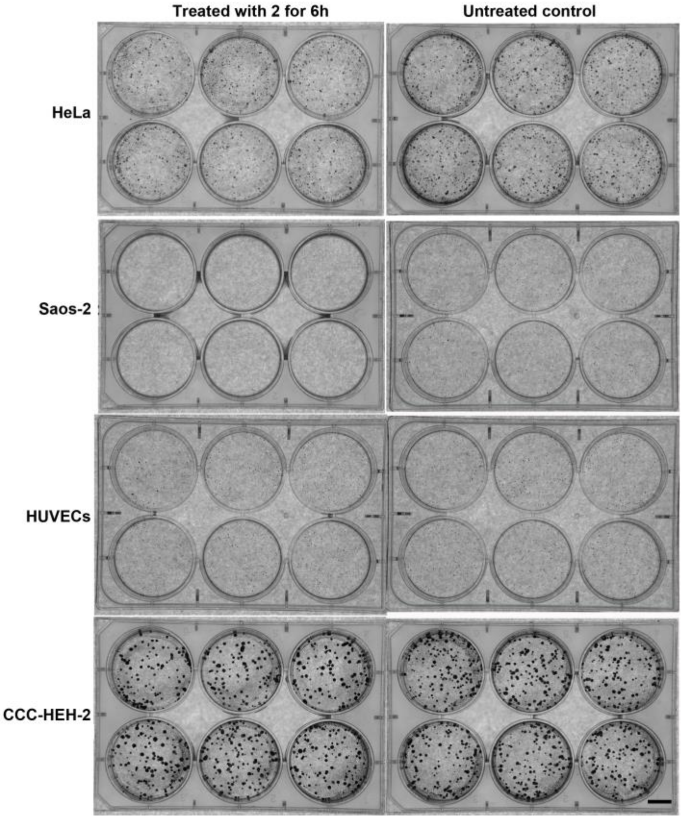

**Supplementary Figure 17. Clonogenic assay of different cell lines before and after treatment with 2.** HeLa cells, HUVECs and Saos-2 cells were seeded at density of 400 cells per well, CCC-HEH-2 cells were seeded at density of 200 cells per well. Cells were incubated with 500 μM **2** for 6 h in the treatment groups. Cells were not treated with compound **2** in the control groups. Colonies were harvest approximately 10 days after seeding. Scale bar, 10 mm.

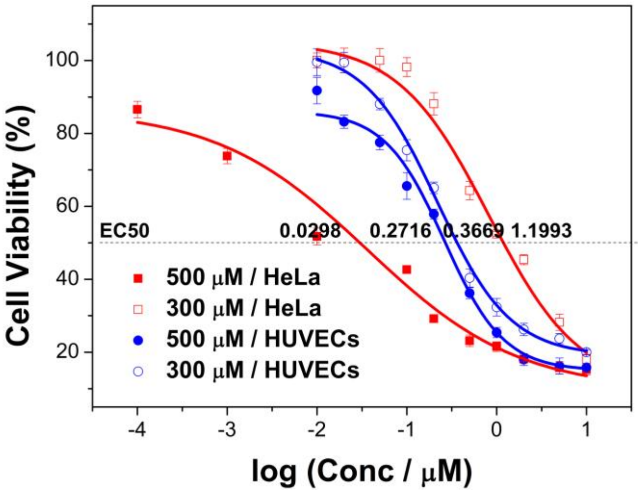


**Supplementary Figure 18. Cytotoxicity of activated Dox against HeLa cells and HUVECs.** HeLa cells or HUVECs at the density of $3x10^3$ cells per well were incubated with 100 μL of pre-warmed medium containing compound **2** at 300 or 500 μM for 6 h. After washing with PBS, 100 μL of 0.0001-10 μM TCO-Dox was added. After 72 h, cell proliferation was assessed by the MTT assay (n= 6). Error bars indicate standard deviation.

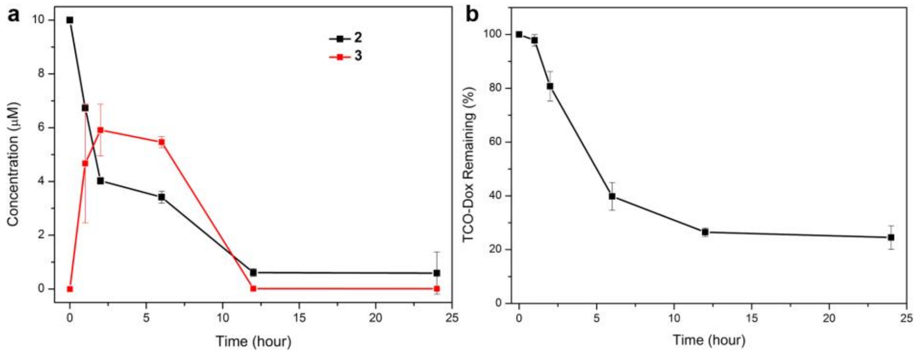


**Supplementary Figure 19. Serum stability study of compound 2 and TCO-Dox. (a)** Compound **2**. **(b)** Compound TCO-Dox**.** 10 μM **2** or TCO-Dox were dissolved in human serum. At each time point, the remaining **2**, corresponding generated **3** and remaining TCO-Dox were determined by UPLC-MS/MS. The decrease of **2** roughly equals to the increase of **3** in the first 6 hours due to dephosphorylation. Both compounds then underwent degradation in serum afterwards (n= 5). Error bars indicate standard deviation.

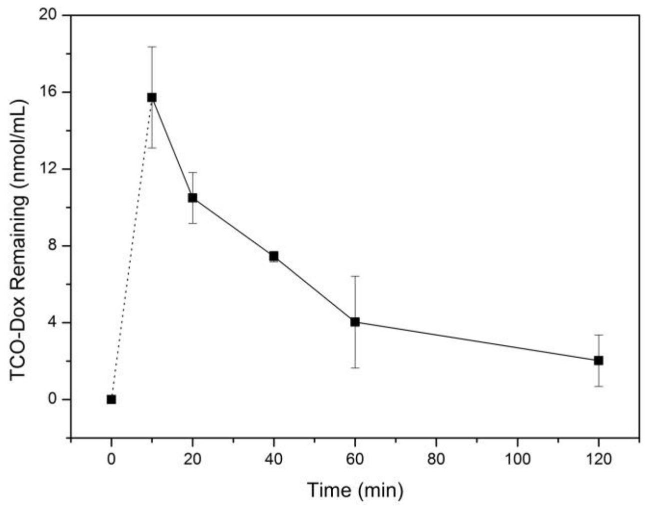


**Supplementary Figure 20. Concentration of TCO-Dox in plasma.** The blood samples from the mice received intravenous injection of 30 mg $kg^{-1}$ TCO-Dox were collected from tail veins at selected time intervals. The remaining TCO-Dox was determined by UPLC-MS/MS (n= 5). Error bars indicate standard deviation.

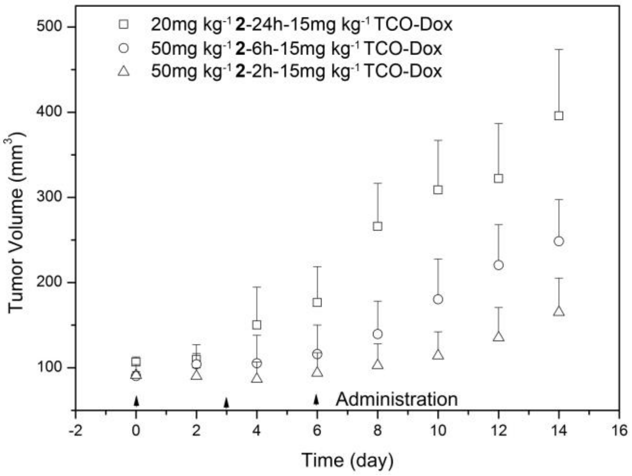


**Supplementary Figure 21. Tumor growth curves of mice after different treatment procedure.** Tumor-bearing mice were receiving the treatment of an intravenous injection of 20 or 50 mg $kg^{-1}$ **2**, followed with an injection of 15 mg $kg^{-1}$ TCO-Dox after 2-24 h interval. The arrows on *x*-axis marked the treatment days (n= 8). Error bars indicate standard deviation.

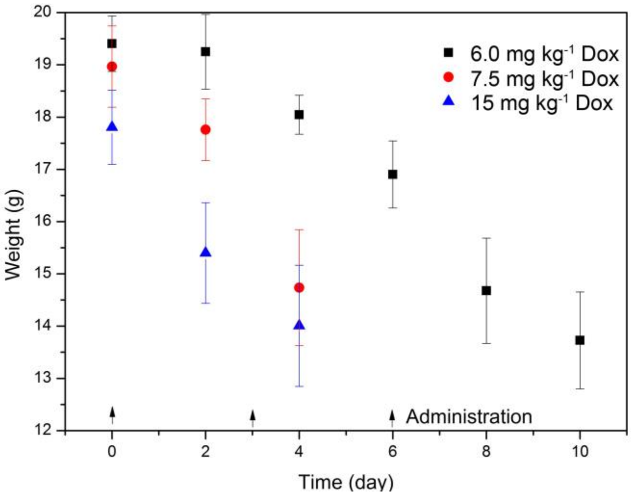


**Supplementary Figure 22. Effect of the dosage of Dox to the mice body weight.** All of the mice died within five days after receiving 15 mg kg$^{-1}$ Dox; the mice began to die six day after receiving 7.5 mg kg$^{-1}$ Dox; the mice began to die eleven day after receiving 6.0 mg kg$^{-1}$ Dox. The arrows on *x*-axis marked the administrations of Dox (n= 8). Error bars indicate standard deviation.

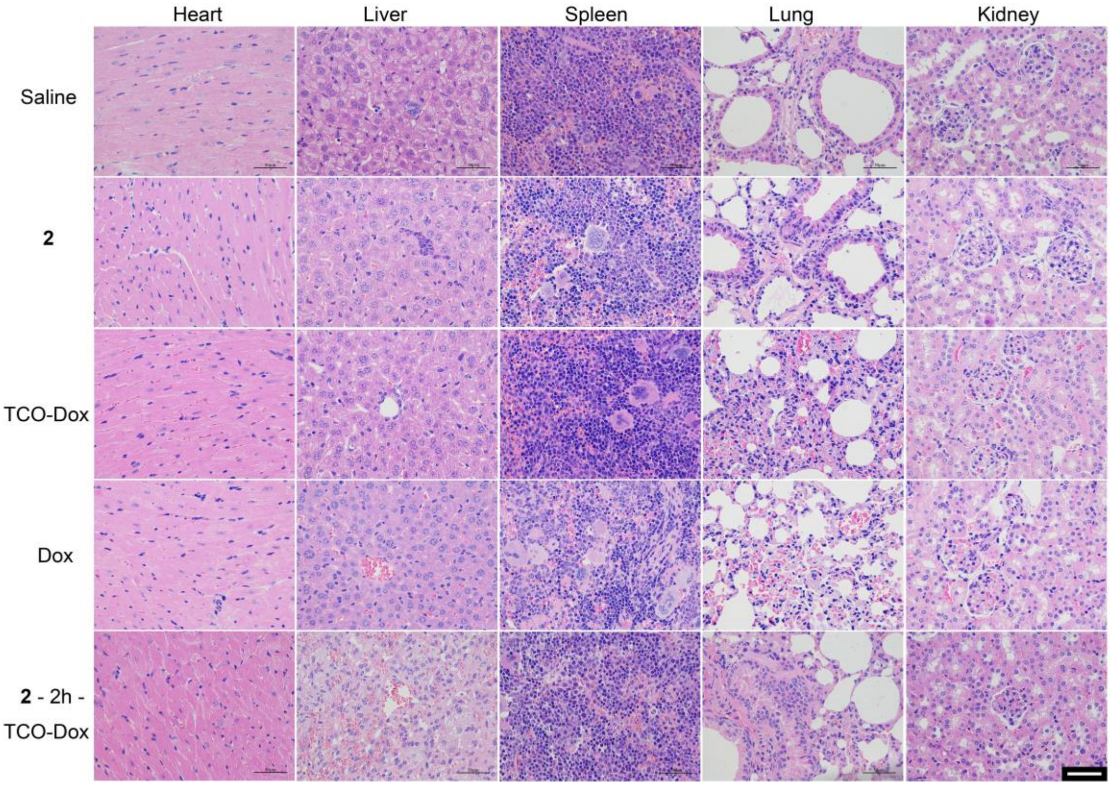


**Supplementary Figure 23. Histological examination of heart, liver, spleen, lung and kidney.** Organs were harvested after treatment at day 14 and stained by hematoxylin and eosin. Tissue biopsy did not show any observable differences between **2**-2 h-TCO-Dox treatment group and the control groups. Scale bar, 50 μm.

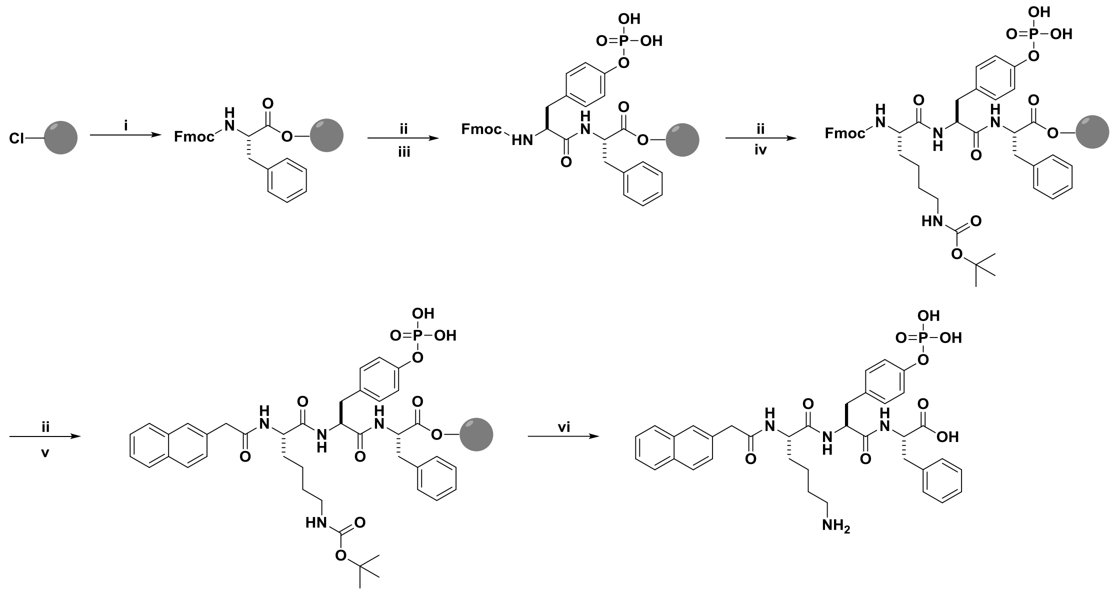


**i) Fmoc-L-Phe-OH, DIPEA, DMF; ii) 20% piperidine / DMF; iii) Fmoc-L-Tyr($PO_3H_2$)-OH, HBTU, DIPEA, DMF;**

**iv) Fmoc-L-Lys(Boc)-OH, HBTU, DIPEA, DMF; v) 2-Naphthylacetic acid, HBTU, DIPEA, DMF; vi) TFA**

**Supplementary Figure 24. Synthetic route and molecular structure of NapKYpF.**

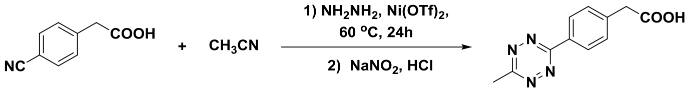


**Supplementary Figure 25. Synthetic route and molecular structure of Tz-COOH.**

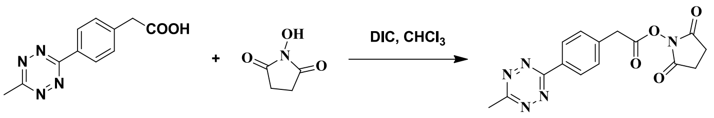


**Supplementary Figure 26. Synthetic route and molecular structure of Tz-NHS.**

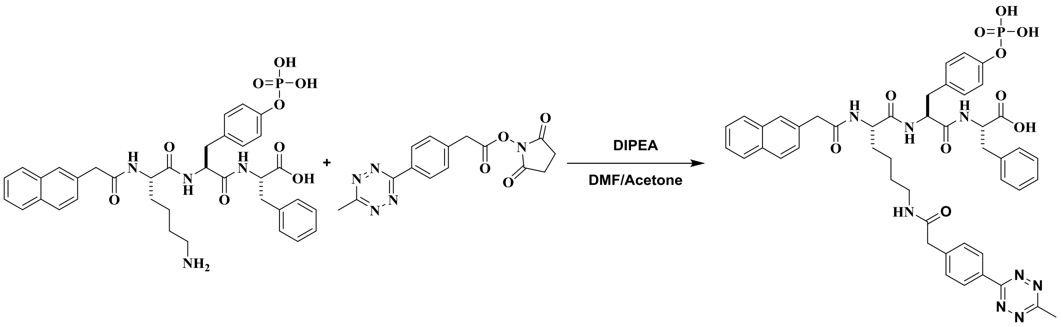


**Supplementary Figure 27. Synthetic route and molecular structure of NapK(Tz)YpF.**

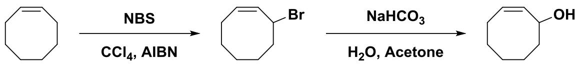


**Supplementary Figure 28. Synthetic route and molecular structure of (Z)-cyclooct-2-en-1-ol.**

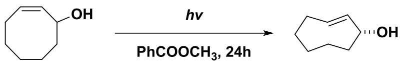


**Supplementary Figure 29. Synthetic route and molecular structure of TCO.**

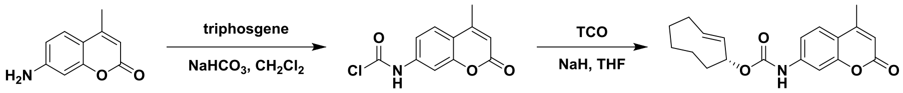


**Supplementary Figure 30. Synthetic route and molecular structure of TCO-CMR.**

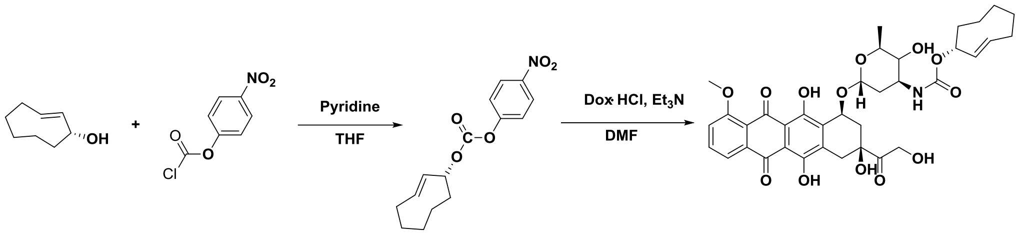


**Supplementary Figure 31. Synthetic route and molecular structure of TCO-Dox.**

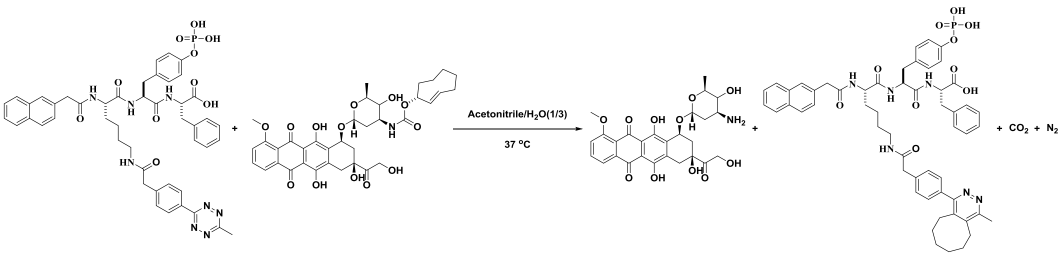


**Supplementary Figure 32. Activation of TCO-Dox *in vitro*.**

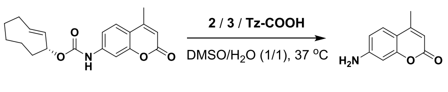


**Supplementary Figure 33. Activation of TCO-Dox *in vitro*.**

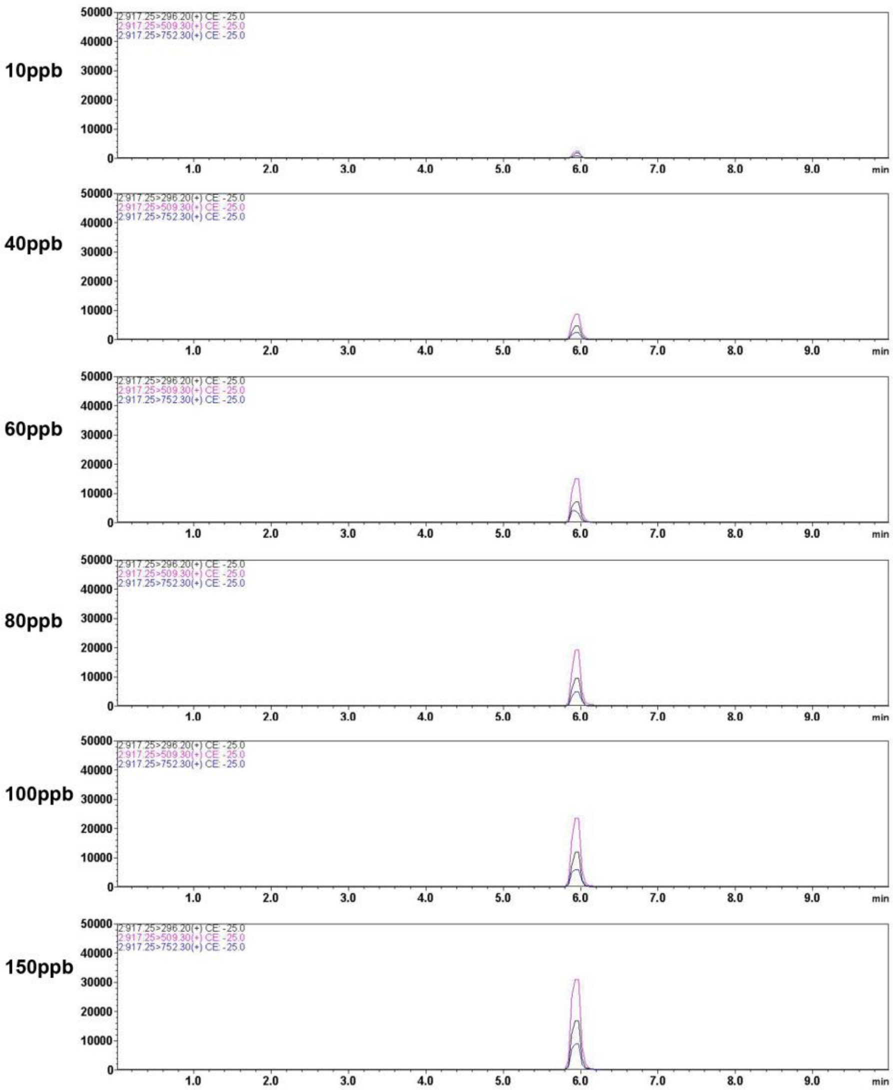


**Supplementary Figure 34. HPLC chromatograms of compound 2.** 2 μL of sample was injected.

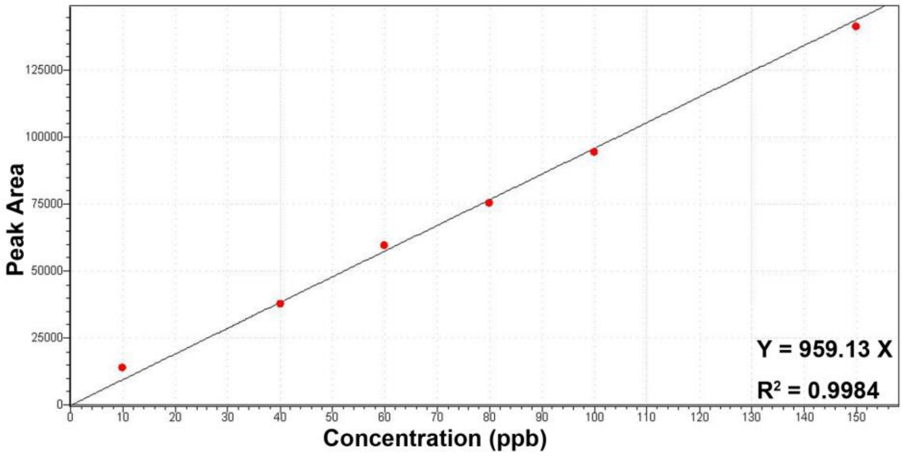


**Supplementary Figure 35. Calibration curve of compound 2.** 2 μL of sample was injected.

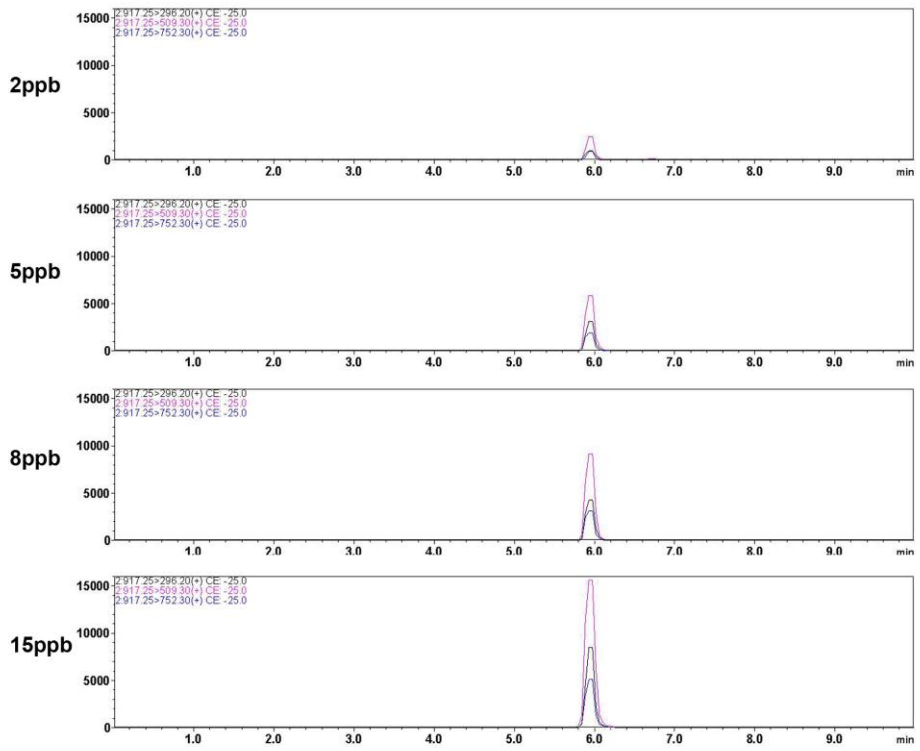


**Supplementary Figure 36. HPLC chromatograms of compound 2.** 10 μL of sample was injected.

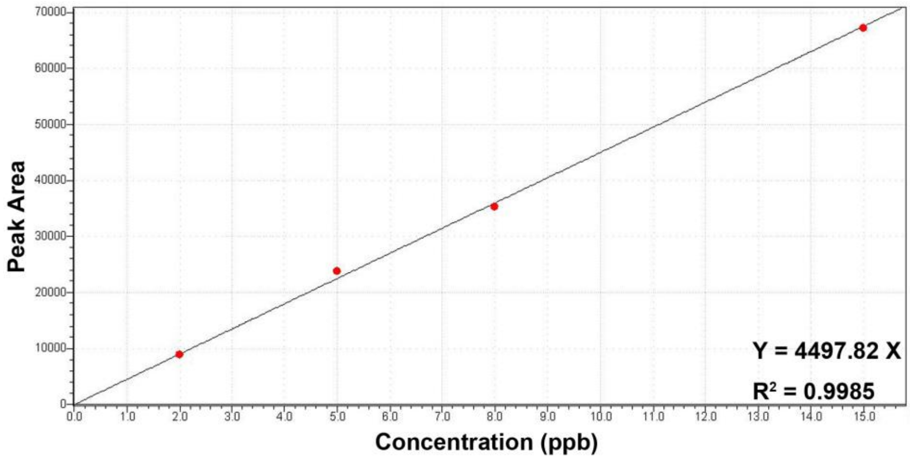


**Supplementary Figure 37**. Calibration curve of compound **2.** 10 μL of sample was injected.

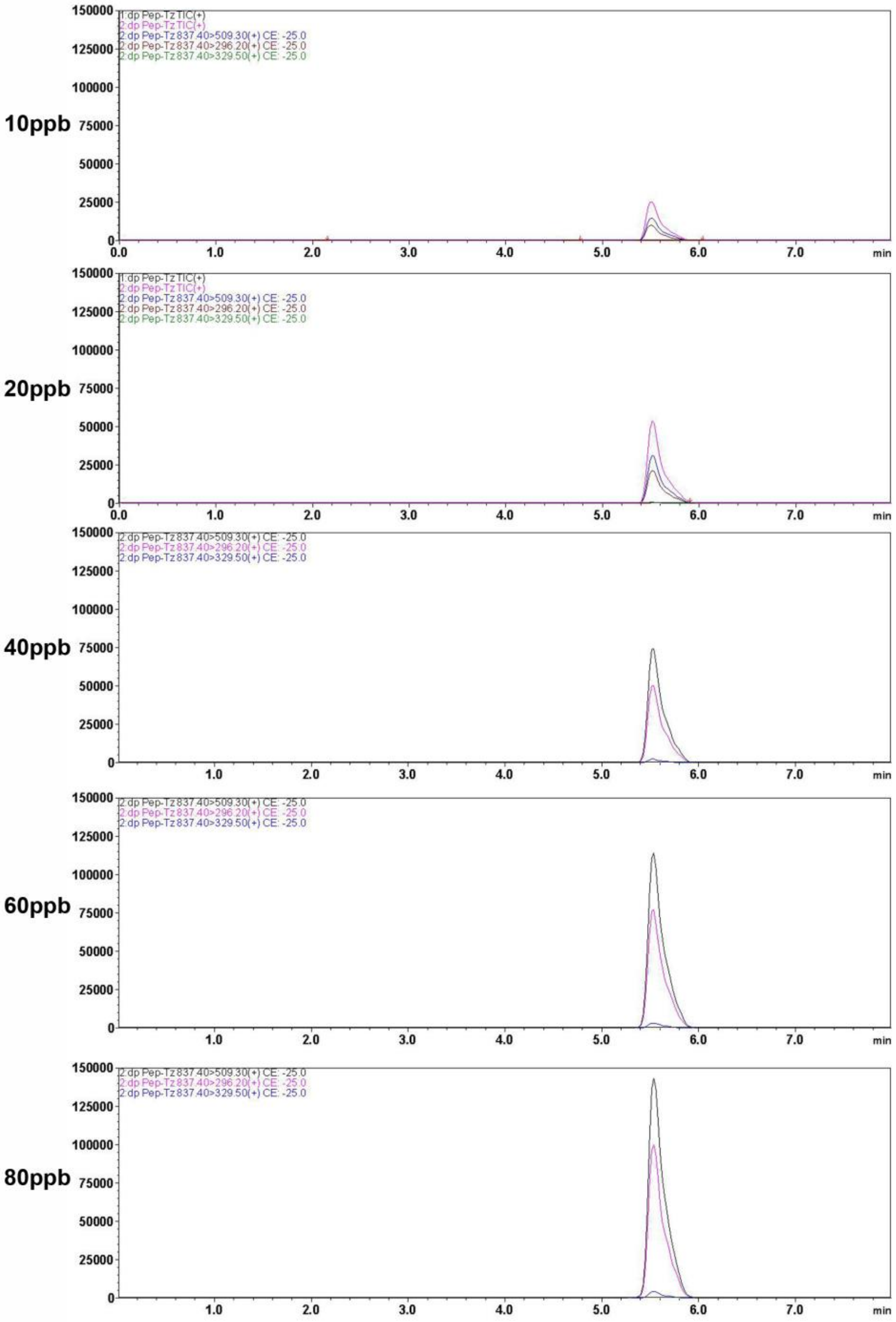


**Supplementary Figure 38. HPLC chromatograms of compound 3.** 10 μL of sample was injected.

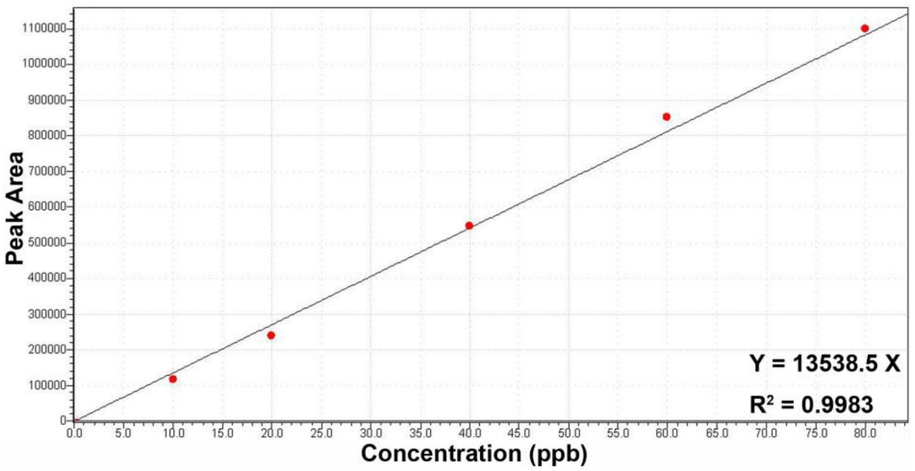


**Supplementary Figure 39. Calibration curve of compound 3.** 10 μL of sample was injected.

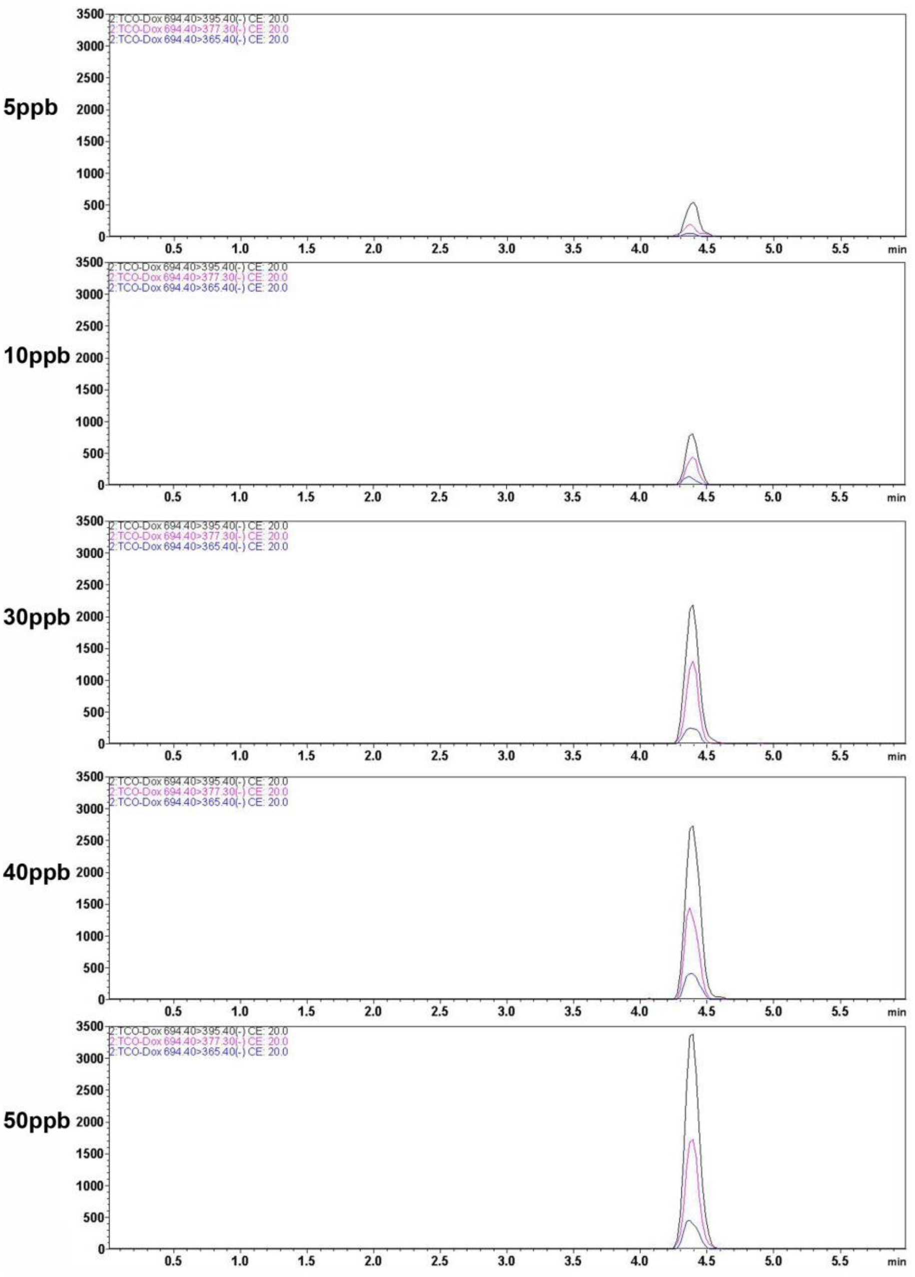

**Supplementary Figure 40. HPLC chromatograms of compound TCO-Dox.** 2 μL of sample was injected.

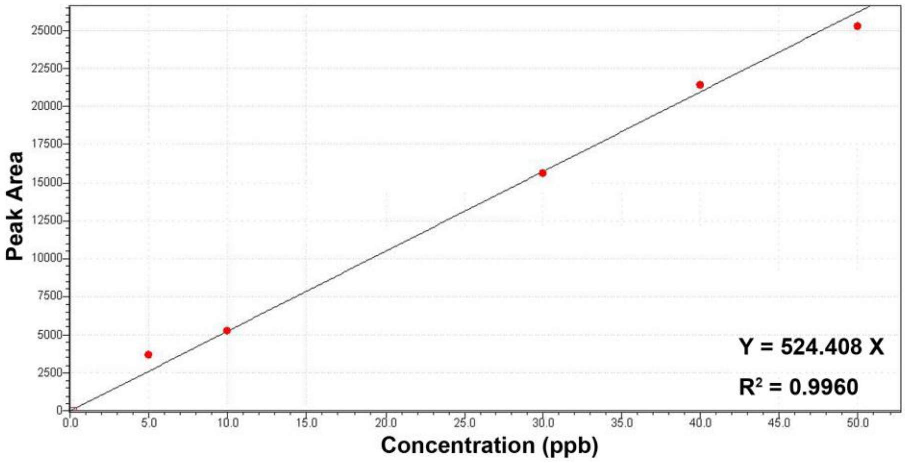


**Supplementary Figure 41. Calibration curve of compound TCO-Dox.** 2 μL of sample was injected.

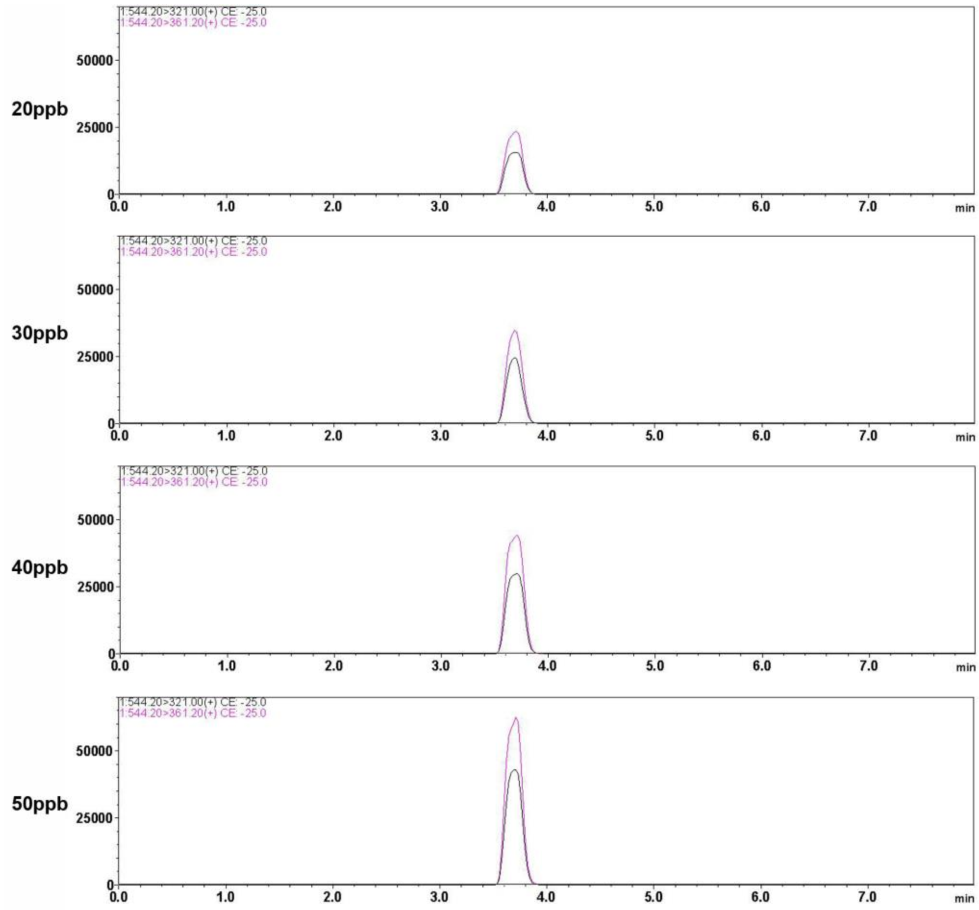


**Supplementary Figure 42. HPLC chromatograms of activated Dox.** 10 μL of sample was injected.

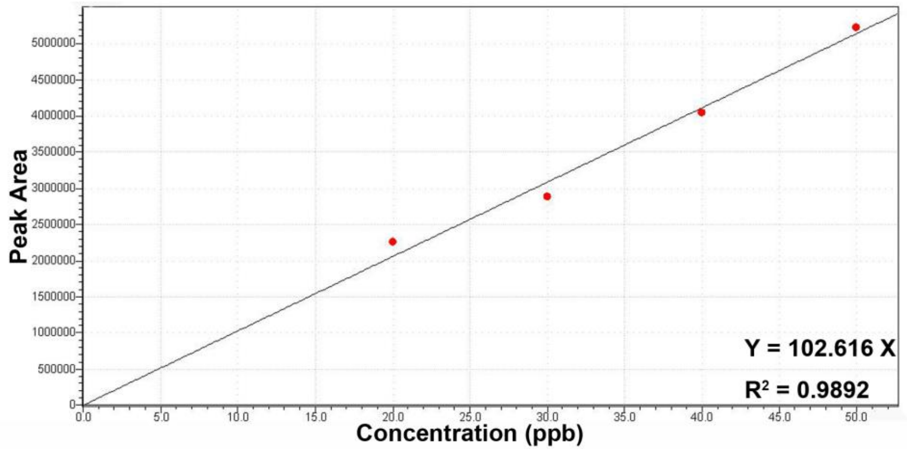


**Supplementary Figure 43. Calibration curve of activated Dox.** 10 μL of sample was injected.

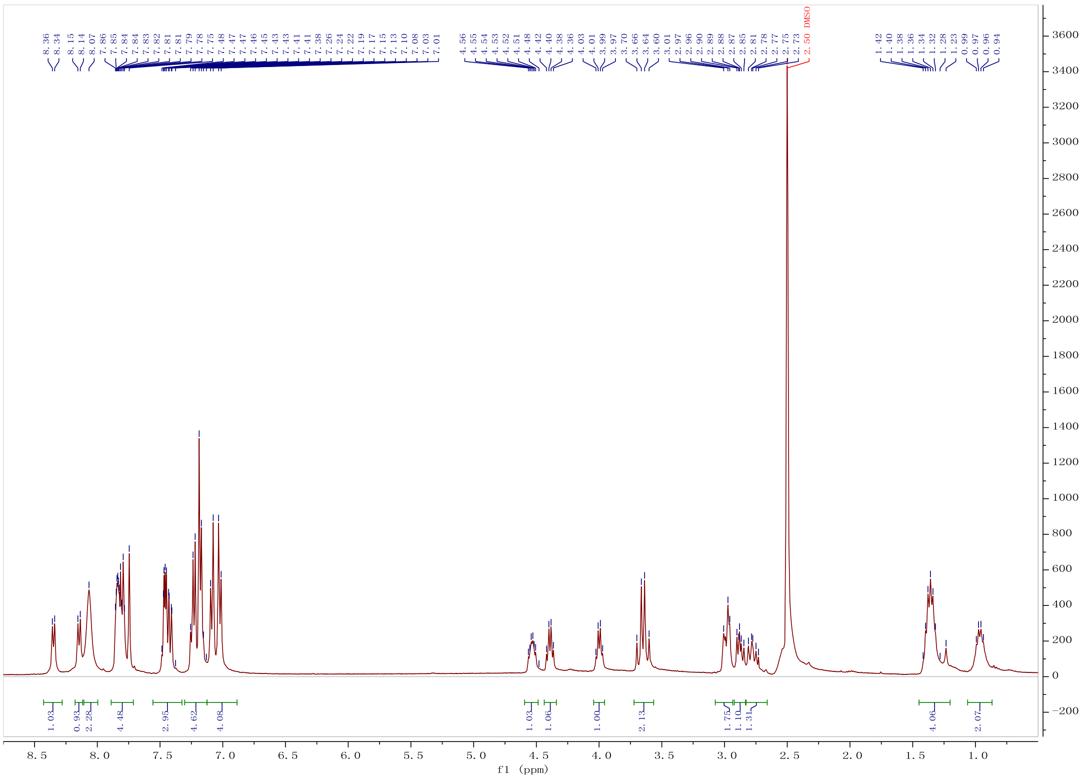


**Supplementary Figure 44. $^1$H NMR spectrum (DMSO-*d6*) of NapKYpF.**

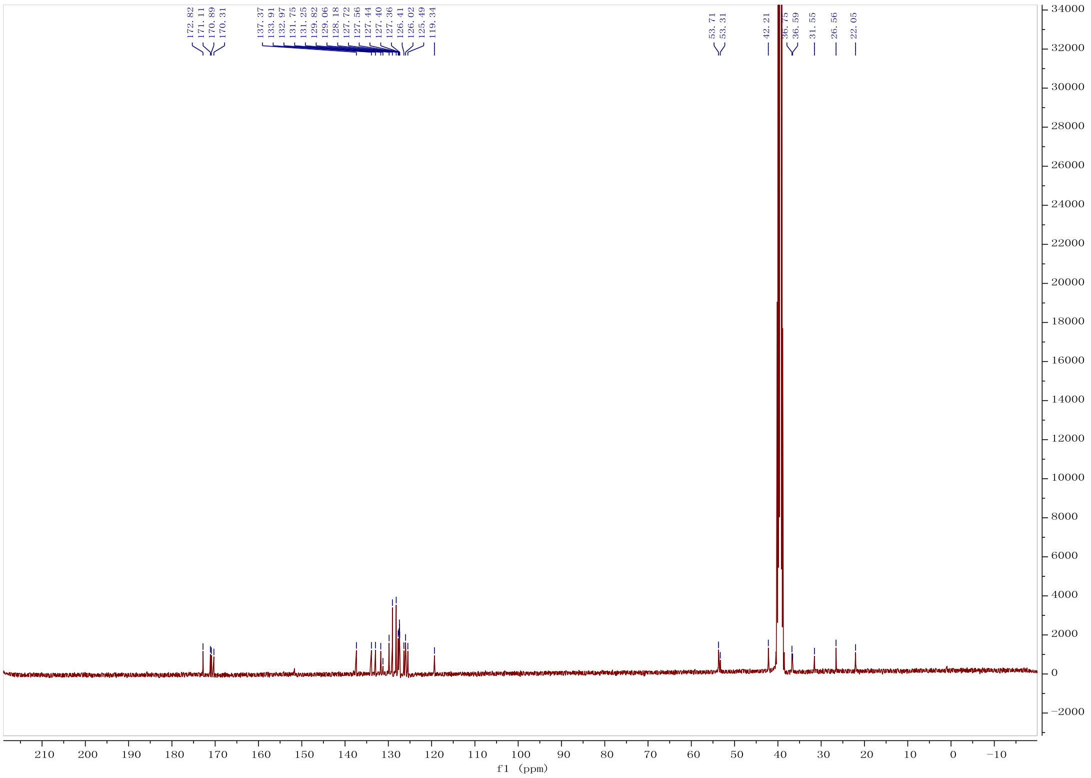


**Supplementary Figure 45.** $^{13}$**C NMR spectrum (DMSO-*d6*) of NapKYpF.**

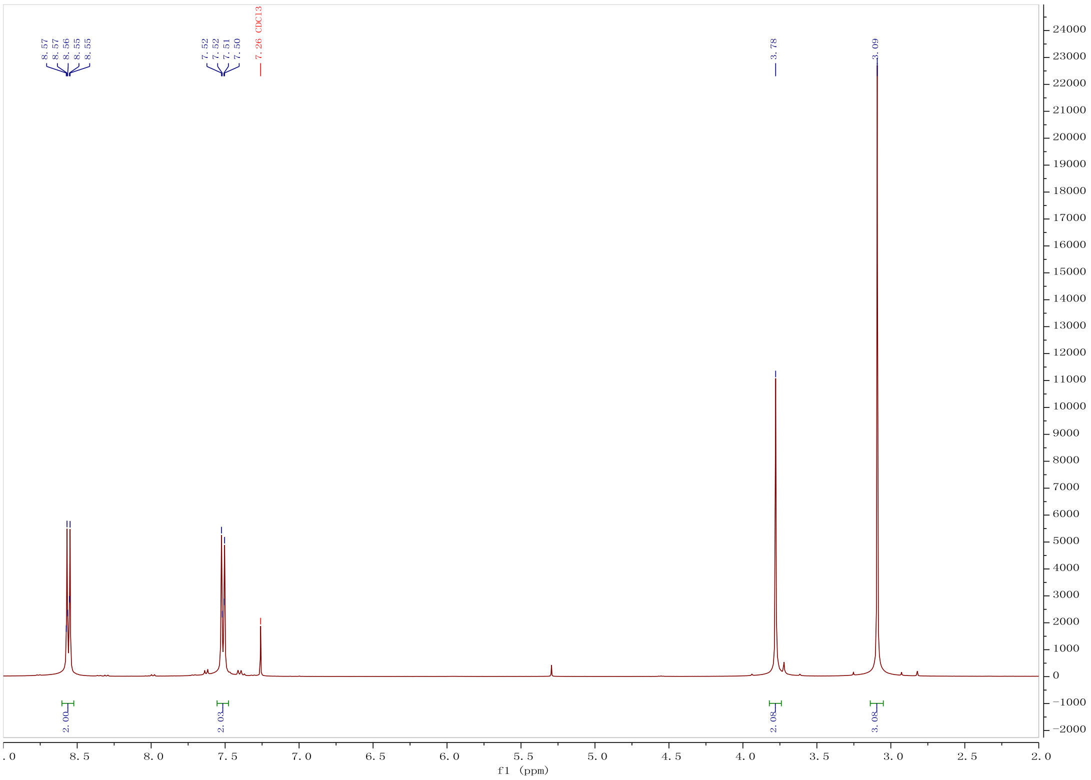


**Supplementary Figure 46.** $^{1}$**H NMR spectrum (**$CDCl_3$**) of Tz-COOH.**

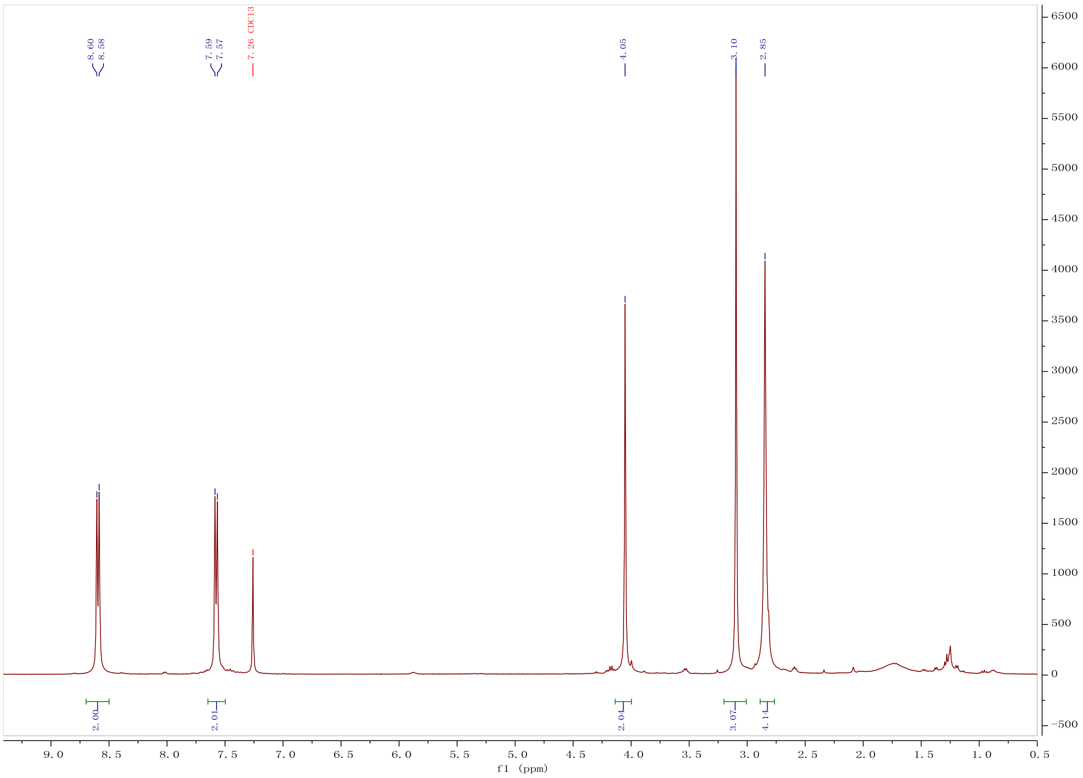

Supplementary Figure 47. $^{1}$H NMR spectrum ($CDCl_3$) of Tz-NHS.

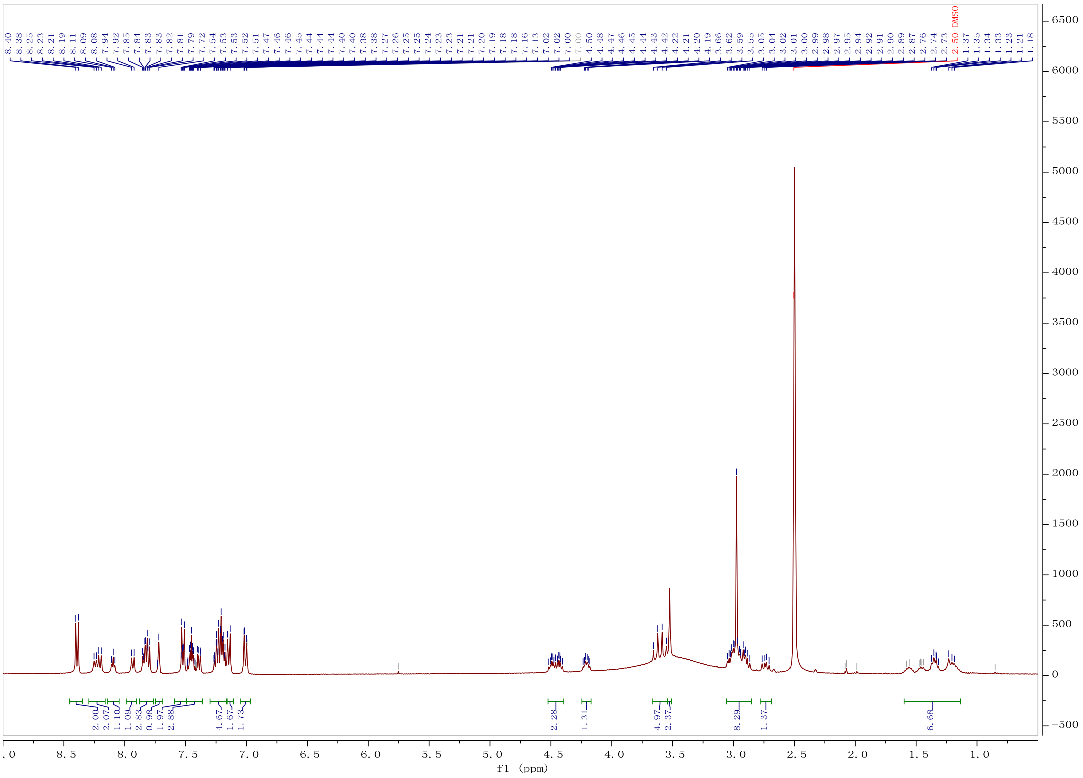

Supplementary Figure 48. $^{1}$H NMR spectrum (DMSO-*d6*) of NapK(Tz)YpF.

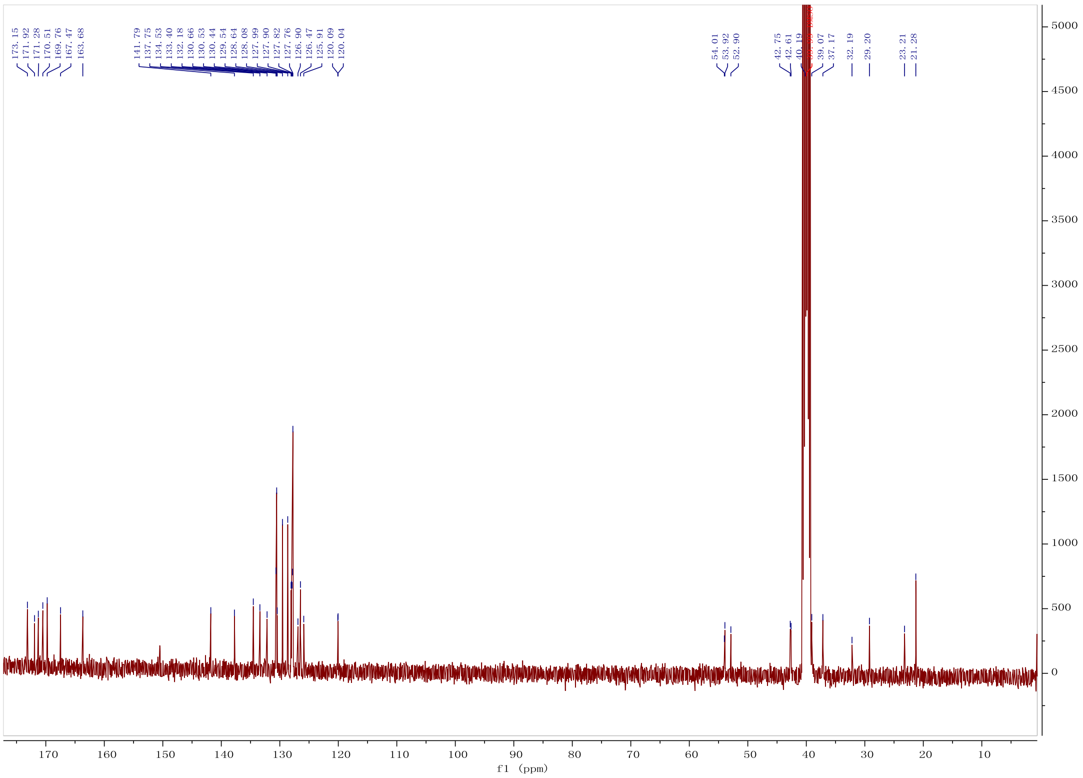

**Supplementary Figure 49. $^{13}$C NMR spectrum (DMSO-*d6*) of NapK(Tz)YpF.**

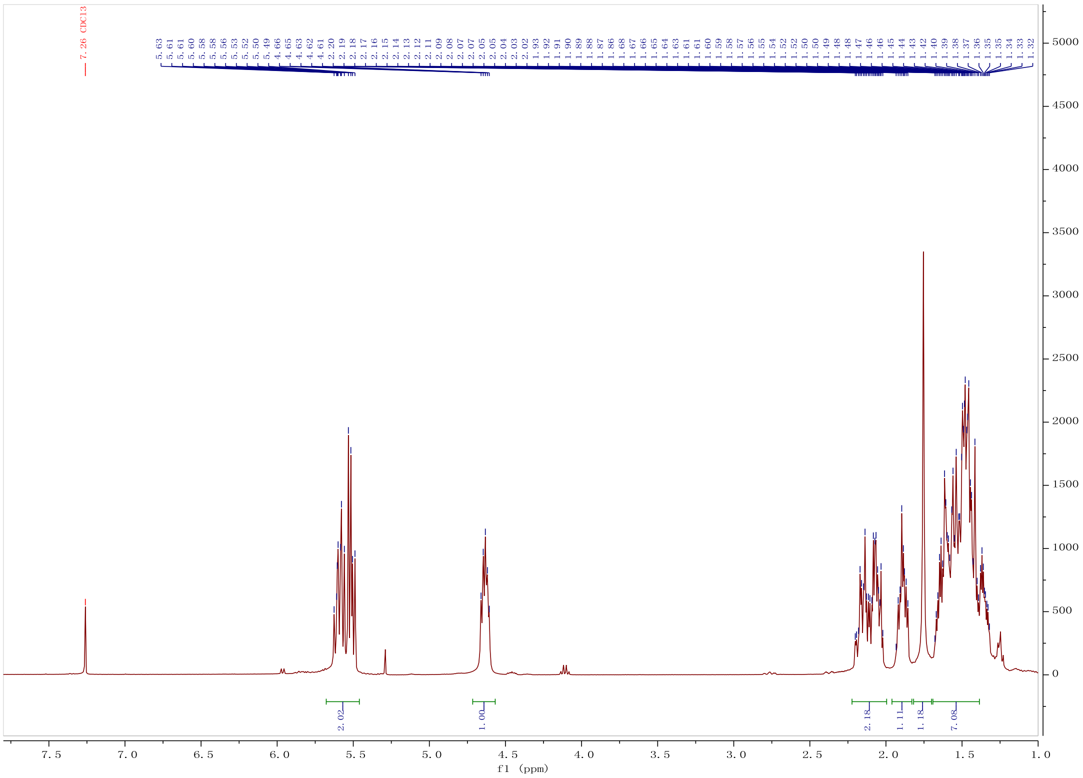

**Supplementary Figure 50. $^{1}$H NMR spectrum ($CDCl_3$) of (Z)-cyclooct-2-en-1-ol.**

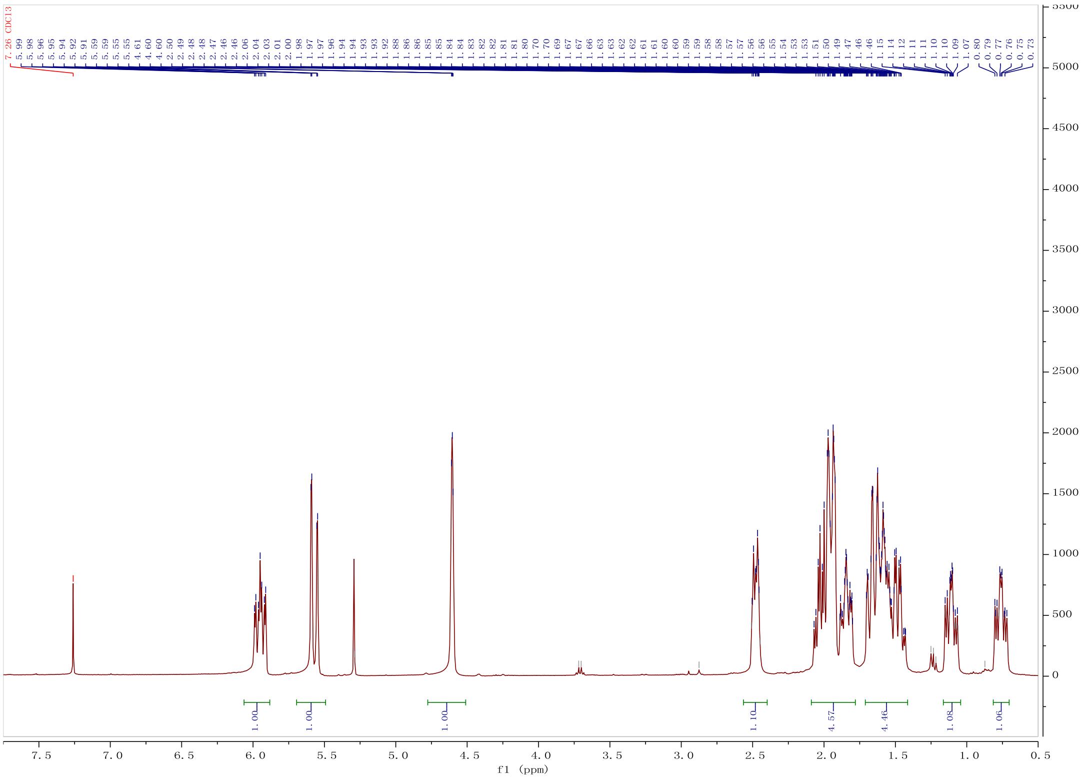


**Supplementary Figure 51. $^{1}$H NMR spectrum ($CDCl_3$) of TCO.**

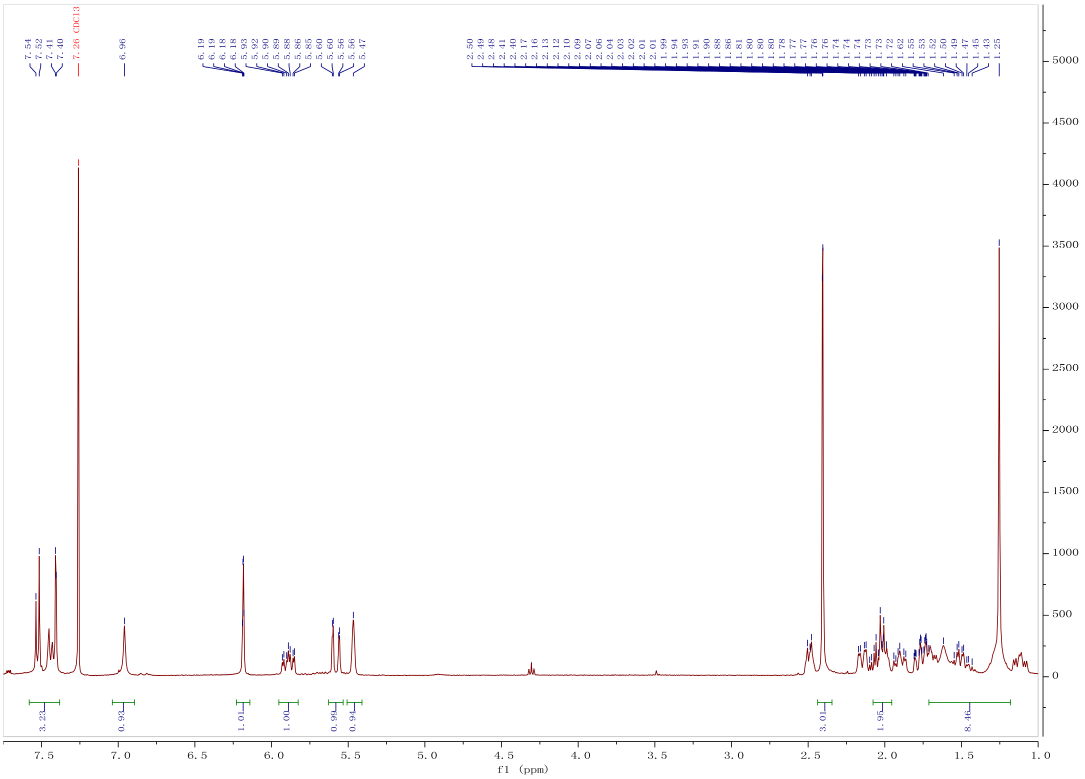


**Supplementary Figure 52. $^{1}$H NMR spectrum ($CDCl_3$) of TCO-CMR.**

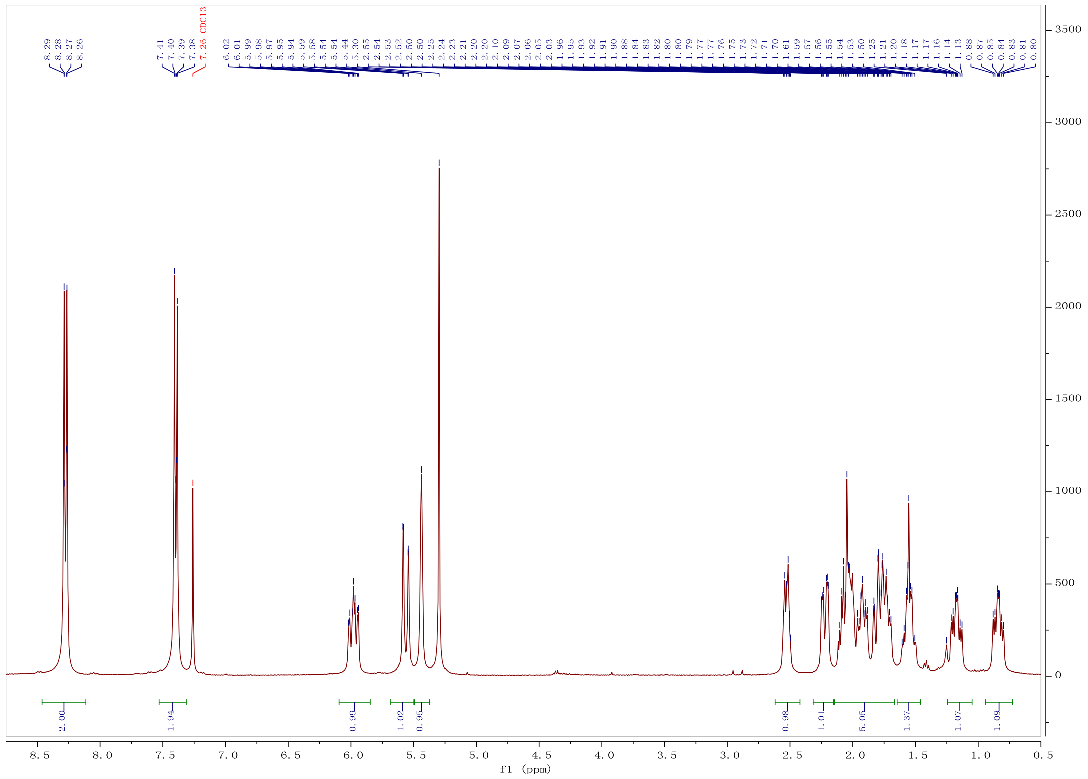

**Supplementary Figure 53. $^1H$ NMR spectrum ($CDCl_3$) of Axial-(E)-cyclooct-2-en-1-yl (4-nitrophenyl) carbonate.**

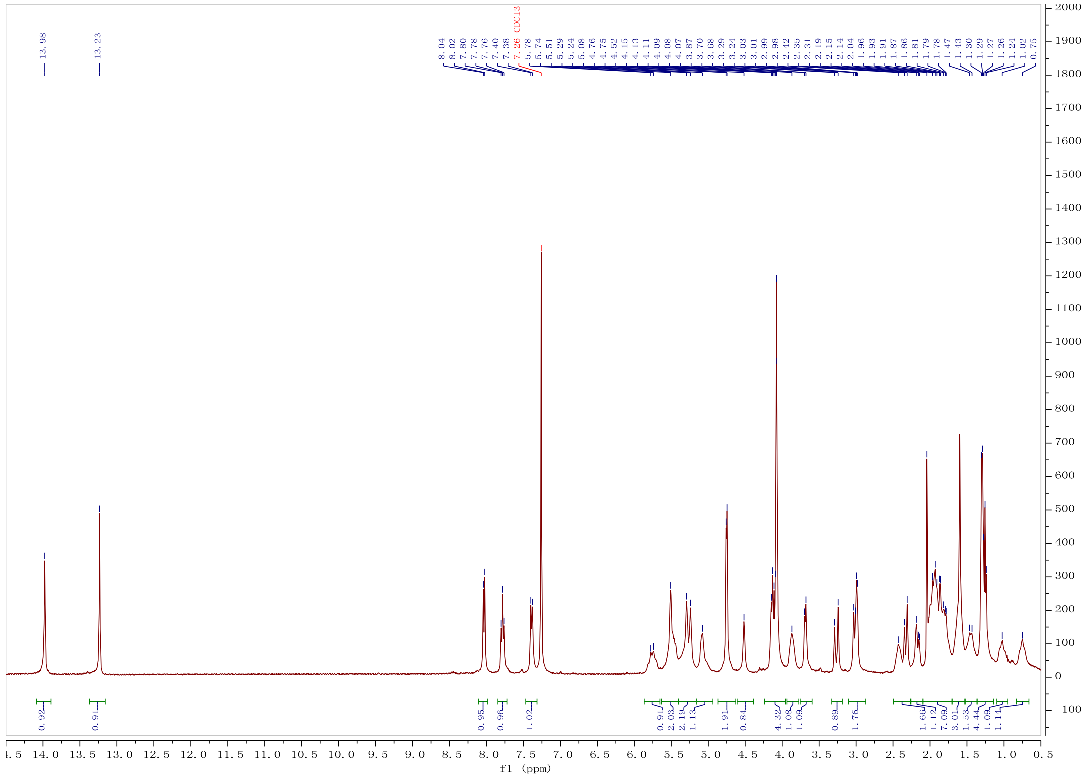

**Supplementary Figure 54. $^1H$ NMR spectrum ($CDCl_3$) of TCO-Dox.**

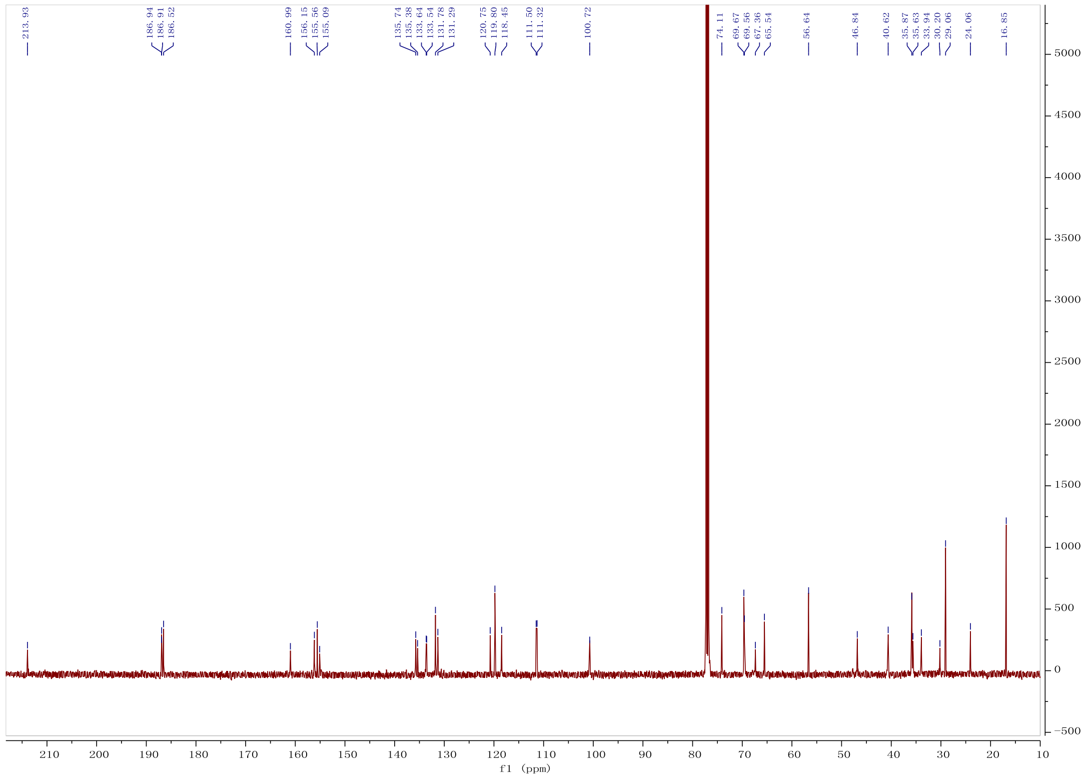


**Supplementary Figure 55. $^{13}C$ NMR spectrum ($CDCl_3$) of TCO-Dox.**

## Supplementary Tables

**Supplementary Table 1. Clonogenic assay on HeLa, HUVECs, Saos-2 and CCC-HEH-s cells pre-treated with 500 μM 2 for 6 h.** The protocol can be found in **Supplementary Methods**.

| Cells | Plating Efficiency | Surviving Fraction |
|---|---|---|
| HeLa | 42.5±3.1% | 75.3±1.9% |
| Saos-2 | 25.5±2.6% | 0±0% |
| HUVECs | 47.4±3.2% | 96.2±3.6% |
| CCC-HEH-2 | 61.8±3.8% | 92.2±8.3% |

## Supplementary Methods

**General Methods:** All chemical reagents and solvents were used as receiving from commercial sources without further purification unless otherwise stated. NMR spectra were recorded on a Bruker 400 MHz Fourier transform spectrometer. TEM images were obtained on a Tecnai G2 20 S-TWIN transmission electron microscope. SPECT/CT was performed on a NanoScan SPECT/CT imaging system (Mediso, Budapest, Hungary). UPLC-MS/MS analyses were performed using a Triple Quadrupole Liquid Chromatograph Mass Spectrometer (LCMS 8050, Shimadzu) in multiple reaction-monitoring mode (MRM). High-resolution ESI mass spectra (HRMS) were recorded on a Q-Exactive LC-MS/MS mass spectrometer. The fluorescent images were captured on a confocal microscope (Zeiss 710). The fluorescence intensity of coumarin was measured on an EnSpire Multimode Plate Reader from PerkinElmer.

**Synthesis of NapKYpF (1):** NapKYpF (**1**) was synthesized *via* a typical solid phase peptide synthesis (SPPS) using 2-chlorotrity resin and the corresponding Fmoc-protected amino acids. The synthetic route and molecular structure of NapKYpF were shown in Supplementary Figure 24. The product was purified by semi-preparative HPLC to give white powder. $^{1}$H NMR (DMSO-*d*6, 400 MHz) $\delta$ (ppm): 8.36 (d, 1H), 8.15 (d, 1H), 8.07 (s, 2H), 7.85-7.75 (m, 4H), 7.46-7.41 (m, 3H), 7.26-7.17 (m, 5H), 7.08-7.01 (q, 4H), 4.61-4.36 (m, 2H), 4.03-3.97 (m, 1H), 3.70-3.60 (q, 2H), 3.01-2.73 (m, 4H), 1.42-1.23 (m, 4H), 0.97-0.94 (m, 2H). $^{13}$C NMR (DMSO-*d*6, 400 MHz) $\delta$ (ppm): 172.82, 171.11, 170.89, 170.31, 137.37, 133.91, 132.97, 131.75, 131.25, 129.82, 129.06, 128.18, 127.72, 127.56, 127.44, 127.40, 127.36, 126.41, 126.02, 125.49, 119.34, 53.71, 53.31, 42.21, 36.75, 36.59, 31.55, 26.56, 22.05. HRMS (m/z): calcd. for $C_{36}H_{41}N_4O_9P$, 704.26; found $[M+H]^+$, 705.2659; $[M-H]^-$, 703.2538.

**Synthesis of 2-(4-(6-methyl-1,2,4,5-tetrazin-3-yl)phenyl) acetic acid:** 2-(4-(6-methyl-1,2,4,5-tetrazin-3-yl)phenyl) acetic acid (Tz-COOH) was prepared according to the reference.[1] The synthetic route and molecular structure of Tz-COOH were shown in Supplementary Figure 25. Acetonitrile (0.52 mL, 10.0 mmol) was added to the mixture of 2-(4-cyanophenyl)ethanoic acid (161.2 mg, 1.0 mmol) and Nickel(II)trifluoromethanesulfonate (178.4 mg, 0.50 mmol) under the atmosphere of $N_2$, and then hydrazine hydrate (1.68 mL, 50.0 mmol) was slowly added. The mixture was stirred in an oil bath at 60 $^oC$ for 24 h. The dark reaction solution was then cooled to room temperature. Sodium nitrite (1.28 g, 20 mmol) dissolved in 10 mL water was slowly added to the reaction mixture followed by slow addition of 1 M HCl until the solution pH value is 3.0. The mixture was extracted with EtOAc (3×60 mL) and the combined organic layer was washed with 1M HCl (3×60 mL), distilled water (3×60 mL), saturated NaCl solution (3×60 mL) and dried over anhydrous $Na_2SO_4$ for 2 h. The EtOAc was removed using rotary evaporation and the residue was purified by column chromatography (Hexane:EtOAc=2:1) to give Tz-COOH as a dark purple crystal (95 mg, 41.3%). The characterization of the product matches with the reported data.[1] $^1H$ NMR ($CDCl_3$, 400 MHz) $\delta$ (ppm): 8.57-8.55 (d, 2H), 7.52-7.50 (d, 2H), 3.78 (s, 2H), 3.09 (s, 3H).

**Synthesis of 2,5-dioxopyrrolidin-1-yl 2-(4-(6-methyl-1,2,4,5-tetrazin-3-yl)phenyl) acetate:** 2,5-dioxopyrrolidin-1-yl2-(4-(6-methyl-1,2,4,5-tetrazin-3-yl)phenyl)acetate (Tz-NHS) was synthesized based on the typical NHS activation chemistry.[2] The synthetic route and molecular structure of Tz-NHS were shown in Supplementary Figure 26. Tz-COOH (46.0 mg, 0.2 mmol) and N-hydroxysuccinimide (23 mg, 0.2 mmol) were dissolved in 10 mL $CHCl_3$. After N,N'-Diisopropylcarbodiimide (41.2 mg, 0.2 mmol) was added into the mixture, the solution was stirred at 25 $^oC$ for 4 h. The solvent was removed using rotary evaporation and the residue was purified by column chromatography (Hexane:EtOAc=4:1) to give a dark purple crystal (52.1 mg, 79.6%). $^1H$ NMR ($CDCl_3$, 400 MHz) $\delta$ (ppm): 8.60-8.58 (d, 2H), 7.59-7.57 (d, 2H), 4.05 (s, 2H), 3.10 (s, 3H), 2.85 (s, 4H). HRMS (m/z): calcd. for $C_{15}H_{13}N_5O_4$, 327.10; found $[M+Na]^+$, 350.0848.

**Synthesis of NapK(Tz)YpF (2):** The synthetic route and molecular structure of NapK(Tz)YpF were shown in Supplementary Figure 27. **1** (35.0 mg, 0.05 mmol) was dissolved in 5 mL DMF and DIPEA (11.35 mg, 0.088 mmol) was added to obtain a well-dispersed solution. Tz-NHS (16.4 mg, 0.05 mmol) dissolved in 3 mL acetone was added into the peptide-containing solution dropwise. The mixture was stirred at room temperature for 12 h and then subjected to semi-preparative HPLC purification to give the light purple powder (30.6 mg, 66.6%). $^1H$ NMR (DMSO-*d*6, 400 MHz) $\delta$ (ppm): 8.40-8.38

(d, 2H), 8.25-8.23 (d, 1H), 8.21-8.19 (d, 1H), 8.09 (m, 1H), 7.94-7.92 (d, 1H), 7.85-7.79 (m, H), 7.72 (s, 1H), 7.54-7.51 (d, 2H), 7.47-7.38 (m, 3H), 7.27-7.16 (m, 5H), 7.15-7.00 (q, 4H), 4.52-4.41 (m, 2H), 4.24-4.18 (m, 1H), 3.66-3.55 (q, 2H), 3.52 (s, 2H), 3.05-2.71 (m, 9H), 1.58-1.18 (m, 6H); $^{13}$C NMR (DMSO-*d*6, 400 MHz) $\delta$ (ppm): 173.15, 171.92, 171.28, 170.51, 169.76, 167.47, 163.68, 141.79, 137.75, 134.53, 133.40, 132.18, 130.66, 130.53, 130.44, 129.54, 128.64, 128.08, 127.99, 127.90, 127.82, 127.76, 126.90, 126.47, 125.91, 120.09, 120.04, 54.01, 53.92, 52.90, 42.75, 42.61, 40.19, 39.07, 37.17, 32.19, 29.20, 23.21, 21.28; HRMS (m/z): calcd. for $C_{47}H_{49}N_8O_{10}P$, 916.33; found $[M-H]^-$, 915.3236.

**Synthesis of (Z)-cyclooct-2-en-1-ol:** (Z)-cyclooct-2-en-1-ol was prepared according to the reference.[3] The synthetic route and molecular structure of (Z)-cyclooct-2-en-1-ol were shown in Supplementary Figure 28. A mixture of (Z)-cyclooctene (10.0 mL, 77 mmol), N-Bromosuccinimide (12.0 g, 67.44 mmol) and azobisisobutyronitrile (AIBN, 8.0 mg, 0.047 mmol) in $CCl_4$ (40 mL) was stirred at 80 $^oC$ for 2 h under the atmosphere of $N_2$. The reaction was cooled at 0 $^oC$ and the precipitate was removed by filtration. The solvent was removed using rotary evaporation to give (Z)-3-bromocyclooctene as light yellow oil. The product was subsequently dissolved in a mixture of acetone (80 mL) and water (40 mL). $NaHCO_3$ (10 g, 120 mmol) was added and the mixture was stirred at 65 $^oC$ for 1 h. The precipitate was removed by filtration and the filtrate was extracted with diethyl ether (3×60 mL). The combined organic layer was dried over anhydrous $Na_2SO_4$ for 2 h and vaporized using rotary evaporation to give brown thick oil. The crude product was purified by column chromatography (Hexane:EtOAc=10:1) to give (Z)-cyclooct-2-en-1-ol as a light yellow oil (7.7 g, 79%). The characterization of the product matches with the reported data.[3] $^1$H NMR ($CDCl_3$, 400 MHz) δ (ppm): 5.63-5.49 (m, 2H), 4.66-4.61 (m, 1H), 2.20-2.02 (m, 2H), 1.91-1.85 (m, 1H), 1.75 (s, 1H), 1.68-1.32 (m, 7H).

**Synthesis of axial-(E)-cyclooct-2-enol:** Axial isomer of (E)-cyclooct-2-enol (TCO) was synthesized according to the reference.[3] The synthetic route and molecular structure of TCO were shown in Supplementary Figure 29. A quartz reaction flask containing (Z)-cyclooct-2-en-1-ol (8.5 g, 67 mmol) and methyl benzoate (9.2 g, 67 mmol) in a mixture of 400 mL diethyl ether and 800 mL petroleum ether was photo irradiated at 254 nm for 24 h. During photo irradiation, the reaction mixture was continuously pumped through a column containing $AgNO_3$ (13.19 g, 77.6 mmol)-impregnated silica gel (117 g). The column was placed in the dark during the reaction. The silica gel was then washed with 1000 mL $CH_2Cl_2$. The silica was collected, stirred in a mixture of aqueous ammonia (500 mL) and $CH_2Cl_2$ (500 mL). The organic layer was separated and the aqueous layer was extracted with $CH_2Cl_2$ (3×200 mL). The combined organic layer was dried over anhydrous

$Na_2SO_4$, and vaporized to give brown thick oil. The crude product was purified by column chromatography (Hexane:EtOAc=100:1 to 75:1) to give TCO (1.43 g, 17.1%) as colorless thick oil. The characterization of the product matches with the reported data.[3] $^1$H NMR ($CDCl_3$, 400 MHz) δ (ppm): 5.99-5.91 (m, 1H), 5.59-5.55 (d, 1H), 4.60 (s, 1H), 2.50-2.45 (m, 1H), 2.07-1.80 (m, 4H), 1.70-1.43 (m, 4H), 1.15-1.06 (m, 1H), 0.80-0.72 (m, 1H).

**Synthesis of TCO-CMR:** TCO-CMR was synthesized according to the reference.[4] The synthetic route and molecular structure of TCO-CMR were shown in Supplementary Figure 30. 7-amino-2-methylcoumarin (CMR, 35 mg, 0.2 mmol) was dissolved in 5 mL $CH_2Cl_2$, followed by adding triphosphogene (98.2 mg , 0.3 mmol) and $NaHCO_3$ (50.4 mg, 0.6 mmol) in the solution. The mixture was stirred at 45 $^oC$ for 4 h. The volatile material was removed under reduced pressure. The residue was dissolved in 1 mL anhydrous THF. The solution was added into the mixture of TCO (25.2 mg, 0.2 mmol) and NaH (2.64 mg, 0.11 mmol) in anhydrous THF under ice-water bath. The mixture was stirred overnight and then water (10 mL) was added dropwise. The mixture was extracted with $CH_2Cl_2$ (3×10 mL). The combined organic phase was dried over anhydrous $Na_2SO_4$ for 2 h. The solvent was removed under reduced pressure and the residual was purified by column chromatography (Hexane:EtOAc=2:1) to give white powder (42 mg, 64.2%). The characterization of the product matches with the reported data.[5] $^1$H NMR ($CDCl_3$, 400 MHz) δ (ppm): 7.54-7.40 (m, 3H), 6.96 (s, 1H), 6.18 (s, 1H), 5.92-5.85 (m, 1H), 5.60-5.56 (d, 1H), 5.47 (s, 1H), 2.41 (s, 3H), 2.01-1.96 (m, 2H), 1.70-1.17 (m, 8H). ESI MS (m/z): calcd. for $C_{19}H_{21}NO_4$, 327.15; found $[M+H]^+$, 328.20.

**Synthesis of axial-(E)-cyclooct-2-en-1-yl doxorubicin carbamate:** The synthetic route and molecular structure of axial-(E)-cyclooct-2-en-1-yl doxorubicin carbamate (TCO-Dox) were shown in Supplementary Figure 31. Pyridine (0.55 g, 7.0 mmol) and 4-nitrophenylchloroformate (0.71 g, 3.5 mmol) was added in a solution of TCO (252 mg, 2 mmol) in 15 mL $CH_2Cl_2$ under ice-water bath. The mixture was stirred overnight. The product was purified by column chromatography (Hexane:EtOAc=10:1) to give (E)-cyclooct-2-en-1-yl (4-nitrophenyl) carbonate as light yellow solid (378.4 mg, 65%). The characterization of the product matches with the reported data.[6] $^1$H NMR ($CDCl_3$, 400 MHz) δ (ppm): 8.29-8.26 (d, 2H), 7.41-7.38 (d, 2H), 6.02-5.94 (m, 1H), 5.59-5.54 (d, 1H), 5.44 (s, 1H), 2.55-2.50 (m, 1H), 2.23-2.20 (d, 1H), 2.12-1.71 (m, 5H), 1.60-1.50 (m, 1H), 1.25-1.13 (m, 1H), 0.88-0.80 (m, 1H).

Axial-(E)-cyclooct-2-en-1-yl doxorubicin carbamate (TCO-Dox) was synthesized according to the reference.[5] (E)-cyclooct-2-en-1-yl (4-nitrophenyl) carbonate (20 mg, 0.0687 mmol) was dissolved in 2 mL DMF. Diisopropylethylamine (80 mg, 0.62 mmol) and doxorubicin hydrochloride (45 mg, 0.0776 mmol) were added in the solution. The mixture

was stirred in the dark at 30 °C for 3 days. 10 mL of water was added. The mixture was extracted with EtOAc (4×20 mL). The combined organic phase was washed with saturated $NaHCO_3$ (3×50 mL), distilled water (3×50 mL), saturated NaCl solution (3×50 mL) and dried over anhydrous $Na_2SO_4$ for 2 h. The solvent was removed under reduced pressure and the residue was purified by column chromatography ($CH_2Cl_2$:MeOH=98:2) to give TCO-Dox (29 mg, 60.6%). The characterization of the product matches with the reported data.[6] $^1$H NMR ($CDCl_3$, 400 MHz) δ (ppm): 13.98 (s, 1H), 13.23 (s, 1H), 8.04-8.02 (d, 1H), 7.78-7.76 (q, 1H), 7.40-7.38 (d,1H), 5.78-5.74 (m, 1H), 5.51 (m, 2H), 5.29-5.24 (d, 2H), 5.08 (s, 1H), 4.76-4.75 (d, 2H), 4.52 (s, 1H), 4.15-4.07 (m, 4H), 3.87 (s, 1H), 3.70-3.68 (d, 1H), 3.29-3.24 (d, 1H), 3.03-2.98 (m, 2H), 2.42-2.31 (m, 2H), 2.19-2.14 (m, 1H), 2.04-1.79 (m, 7H), 1.60 (s, 3H), 1.47-1.43 (m, 1H), 1.30-1.26 (m, 4H), 1.02 (m, 1H), 0.75 (m, 1H). $^{13}$C NMR ($CDCl_3$, 500 MHz) *δ* (ppm): 213.93, 186.94, 186.91, 186.52, 160.99, 156.15, 155.56, 155.09, 135.74, 135.38, 133.64, 133.54, 131.78, 131.29, 120.75, 119.80, 118.45, 111.50, 111.32, 100.72, 74.11, 69.67, 69.56, 67.36, 65.54, 56.64, 46.84, 40.62, 35.87, 35.63, 33.94, 30.20, 29.06, 24.06, 16.85; HRMS (m/z): calcd. for $C_{36}H_{41}NO_{13}$, 695.26; Found $[M-H]^-$: 694.2499.

**Hydrogel preparation:** By adding 10 U $mL^{-1}$ of alkaline phosphatase (ALP) into a solution of 1.0 mg $mL^{-1}$ of **2** in PBS buffer, a transparent and stable hydrogel of **3** was formed. Hydrogel at other concentrations were prepared following the same protocol with different concentrations of **2** accordingly.

**Activation of TCO-Dox:** The activation of TCO-Dox was shown in Supplementary Figure 32. 2.5 μL 20 mM of **2** was mixed with 96.5 μL acetonitrile and 300 μL $H_2O$. After the solution was equilibrated at 37 $^oC$, 2 μL 10 mM of TCO-Dox was added and the solution was thoroughly mixed and incubated at 37 $^oC$ in the dark. The reaction progress was monitored by UPLC-MS/PDA analysis, revealing the formation of the TCO adduct with m/z= +998.69 Da $(M+H)^+$ and showing release of doxorubicin with m/z= +545.03 Da $(M+H)^+$.

**Restoration of fluorescence of coumarin**: The activation of TCO-CMR was shown in Supplementary Figure 33. 1.25 μL 20 mM of tetrazine derivatives was mixed with 97.75 μL DMSO and 100 μL $H_2O$ (For **3**, additional ALP at 10 U $mL^{-1}$ was added to convert **2** to **3**). After the solution was equilibrated at 37 $^oC$, 1 μL 10 mM of TCO-CMR was added and the fluorescence intensity of coumarin was measured on a plate reader (Perkin Elmer) at given time points (ex. 380 nm, em. 450 nm).

**Confocal imaging:** Cells were firstly placed in glass chamber and cultured with 2 mL

culture medium containing 500 μM of **2** for 6 h. Then, the cells were rinsed by PBS and incubated with pre-warmed staining solution (500 nM of ER-Tracker™ Red dyes) for 30 min at 37 $^{o}$C. After rinsing the cells by PBS buffer for 5 times, the cell-containing glass chamber was fixed on the confocal microscope stage. TCO-CMR (1 mL 50 μM dissolved in PBS buffer) was added to the cells that were immediately subject to fluorescent imaging.

**Prodrug activation efficacy in cells:** Cells were plated on flat bottom 96-well plates at a density of 3x10$^3$ cells per well. After 12 h attachment, cells were incubated with 100 μL 500 μM of **2** for 6 h before removing **2** and the addition of 200 μL 0.0001-10 μM of TCO-Dox. Cells were further incubated for 72 h before measuring the viability by the MTT assay.

**Clonogenic assay of cells *in vitro*:** Clonogenic assay was performed according to the standard protocol.[6] 2×10$^5$ attached cells were treated with 500 μM of **2** for 6 h in culture dishes. Cells were then detached, re-suspended in cultural medium and counted accurately. The cell suspension was diluted into the desired seeding concentration and cells were seeded in 6-well plates at a density of 200 cells per well for CCC-HEH-2 and 400 cells per well for HeLa, Saos-2 and HUVECs. After incubation for 10 days, the cultural medium was removed and cells were rinsed carefully with PBS. 2 mL of a mixture of 6.0% (vol/vol) glutaraldehyde and 0.5% (wt/vol) crystal violet was added and kept for 30 min. The glutaraldehyde crystal violet mixture was removed carefully. The colonies were rinsed with water and dried in normal air at room temperature. The number of the colonies was counted using a stereomicroscope.

When untreated cells are plated as a single-cell suspension at low densities, they will grow to colonies. The plating efficiency (PE) is the ratio of the number of colonies to the number of cells seeded:

$$\mathrm{PE} = \frac{\textit{no.}\ \text{of colonies formed}}{\textit{no.}\ \text{of cells seeded}} \times 100\% \qquad \text{(Supplementary Equation 1)}$$

The number of colonies that arise after treatment of cells, expressed in terms of PE, is called the surviving fraction (SF):

$$\mathrm{SF} = \frac{\textit{no.}\ \text{of colonies formed after treatment}}{\textit{no.}\ \text{of cells seeded} \times \mathrm{PE}} \times 100\% \qquad \text{(Supplementary Equation 2)}$$

**SPECT/CT imaging:** The tumor-bearing mice were used for SPECT/CT imaging once the tumor volume reached around 200 mm$^3$. For preparation of $^{125}$I labeled **2**, 60 μg of **2** was dissolved in 150 μL phosphate buffer (pH=7.0) in a vial coated with 20 μg of Iodogen

(Sigma, St. Louis, MO). Then, 9 μL $Na^{125}I$ (2.4 mCi) (Beijing Atom High Tech, Beijing, China) was added and the mixture was kept at room temperature for 1 h until the labeling ratio reached to more than 95%. For SPECT/CT imaging, a mixture of 50 mg $kg^{-1}$ **2** and 0.875 mg $kg^{-1}$ $^{125}I$ labelled **2** (700 μCi) were intravenously administrated to investigate the distribution of **2/3** and 0.875 mg $kg^{-1}$ $^{125}I$ labelled **2** (700 μCi) was administrated as a control. After anesthetization with 2% isoflurane in oxygen, SPECT and helical CT scans of the mice were performed at 2, 5, 7 and 25 h on a NanoScan SPECT/CT imaging system (Mediso, Budapest, Hungary).

**UPLC-MS/MS analyses:** UPLC-MS/MS metabolomic analyses were performed using a Triple Quadrupole Liquid Chromatograph Mass Spectrometer (LCMS 8050, Shimadzu) in multiple reaction-monitoring mode (MRM). Data acquisition and processing were performed using LabSolutions version 5 software. Measurements were carried out at 40°C. The mobile phase for each compound was as follows: (1) for compound **2**, **3** and Dox, solvent A consisted of 0.1% formic acid in water and solvent B consisted of 0.1% formic acid in mass spectrometric grade acetonitrile; (2) for compound TCO-Dox, solvent A consisted of 100% water and solvent B consisted of 100% mass spectrometric grade acetonitrile. Injection volume was 2 or 10 μL. The mass spectrometer was interfaced with the liquid chromatograph using an electrospray ion source. The nitrogen nebulizing gas flow was set at 10 L $min^{-1}$ and the drying gas flow at 15 mL $min^{-1}$. 4500 V were used for the interface voltage. The temperature of the block heater was maintained at 400 °C and the one of the desolvation line at 250 °C. The MRM transitions were 917.25→752.3/509.3/296.2, 873.4→509.3/329.5/296.2, 694.4→395.4/377.3/365.4, 544.2→361.2/321.0, respectively for compound **2**, **3**, TCO-Dox and Dox. The collision energy was -25 V, -25 V, -25 V and 20 V, respectively for compound **2**, **3**, TCO-Dox and Dox. The collision gas used was argon at 230 kPa. The dwell time was set to 100 msec and the pause time to 3 msec.

**Plasmatic stability of compound 2 and TCO-Dox:** To evaluate the stability of **2** and TCO-Dox under physiologically relevant conditions, compounds were incubated in human serum and the amount of the remaining intact compound (the corresponding dephosphorylated **3** was detected as well) was determined by UPLC-MS/MS. Normal human serum was used according to approved guidelines. Stock solutions of each compound were prepared in PBS at a concentration of 5 mM. The stock solution was diluted to 10 μM in human serum. After incubation for various time points (1, 2, 6, 12 and 24 h), 50 μL of the compound-containing serum samples were mixed with 200 μL of cold acetonitrile for compound extraction. After centrifugation at 1559 g for 7 min and filtration, 2 μL of the supernatant was analyzed by UPLC-MS/MS (n=5).

**UPLC-MS/MS analyses of compound 2, 3 and activated Dox in blood samples:** Sample preparation: blood samples were collected from the tail vein of mice. Plasma samples were first prepared by centrifugation at 1559 g for 2 min (4°C), minimum 10 min after blood collection. 90 μL acetonitrile were added per 10 μL of plasma samples which were centrifuged at 1559 g for 7 min to remove proteins. 10 μL of supernatants were then analyzed by UPLC-MS/MS.

**UPLC-MS/MS analyses of compound 2, 3 and activated Dox in tumor and liver samples:** Tumor and liver samples preparation: organs were comminuted in a mixture of water (1 mL) and acetonitrile (1 mL). The comminuted samples were vortexed, sonicated for 5 min and centrifuged at 1559 g for 5 min (4°C). A 2 μL aliquot of supernatants was then analyzed by UPLC-MS/MS for compound **2**, while a 10 μL aliquot of the same supernatants was analyzed by UPLC-MS/MS for compound **3** as well as activated Dox.

## Supplementary References


1. Yang, J., Karver, M. R., Li, W. L., Sahu, S. & Devaraj, N. K. Metal-catalyzed one-pot synthesis of tetrazines directly from aliphatic nitriles and hydrazine. *Angew. Chem. Int. Ed.* **124,** 5312 (2012).
2. Gao, Y., Kuang, Y., Guo, Z. F., Krauss, I. J. & Xu, B. Enzyme-instructed molecular self-assembly confers nanofibers and a supramolecular hydrogel of taxol derivative. *J. Am. Chem. Soc.* **131,** 13576 (2009).
3. Li, J., Jia, S. & Chen, P. R. Diels-Alder reaction-triggered bioorthogonal protein decaging in living cells. *Nat. Chem. Biol.* **10,** 1003-1005 (2014).
4. Fan, X. Y. *et al.* Optimized tetrazine derivatives for rapid bioorthogonal decaging in living cells. *Angew. Chem. Int. Ed.* **55,** 14046-14050 (2016).
5. Versteegen, R. M., Rossin, R., ten Hoeve, W., Janssen, H. M. & Robillard, M. S. Click to release: instantaneous doxorubicin elimination upon tetrazine ligation. *Angew. Chem. Int. Ed.* **52,** 14112 (2013).
6. Franken, N. A. P., Rodermond, H. M., Stap, J., Haveman, J. & van Bree, C. Clonogenic assay of cells in vitro. *Nat. Protoc.* **1,** 2315-2319 (2006).
7. Gao, Y. *et al.* Probing nanoscale self-assembly of nonfluorescent small molecules inside live mammalian cells. *ACS Nano* **7,** 9055-9063 (2013).
