# Supplementary material for: Synergistic enzymatic and bioorthogonal reactions for selective prodrug activation in living systems
Source: Nat Commun. 2018 Nov 28;9:5032. doi: 10.1038/s41467-018-07490-6 (PMC6261997; doi:10.1038/s41467-018-07490-6)
Supplement: Supplementary file 3 — Description of Additional Supplementary Files [file 41467_2018_7490_MOESM3_ESM.pdf]

Description of Additional Supplementary Files

Supplementary Movie 1

Description: The development of fluorescence of coumarin in HeLa cells. The development of bright fluorescent of coumarin (blue) appeared near the nucleus after the addition of 50 μM TCO-CMR to HeLa cells that were pre-incubated with 500 μM **2** for 6 h, indicating the abundance of accumulated tetrazine triggers inside HeLa cells and revealing the high reactivity of the accumulated triggers to activate TCO modified prodrugs.

Supplementary Movie 2

Description: The development of fluorescence of coumarin in HeLa cells stained with ER tracker. The development of blue fluorescence of coumarin colocalized well with the ER tracker (red), which implied the occurrence of EISA on the ER.

Supplementary Movie 3

Description: Z stacks of HeLa cells with liberated coumarin. The development of blue fluorescence of coumarin was near the nucleus.

Supplementary Movie 4

Description: The development of weak fluorescence of coumarin in HUVECs. Weak fluorescence of coumarin (blue) was developed in HUVECs pre-incubated with 500 μM **2** for 6 h, which indicated EISA barely occurred in the HUVECs.
